# AYbRAH: a curated ortholog database for yeasts and fungi spanning 600 million years of evolution

**DOI:** 10.1093/database/baz022

**Published:** 2019-03-20

**Authors:** Kevin Correia, Shi M Yu, Radhakrishnan Mahadevan

**Affiliations:** 1 Department of Chemical Engineering and Applied Chemistry, University of Toronto, College Street, Toronto, ON, Canada; 2 Institute of Biomaterials and Biomedical Engineering, University of Toronto, College Street, Toronto, ON, Canada

## Abstract

Budding yeasts inhabit a range of environments by exploiting various metabolic traits. The genetic bases for these traits are mostly unknown, preventing their addition or removal in a chassis organism for metabolic engineering. Insight into the evolution of orthologs, paralogs and xenologs in the yeast pan-genome can help bridge these genotypes; however, existing phylogenomic databases do not span diverse yeasts, and sometimes cannot distinguish between these homologs. To help understand the molecular evolution of these traits in yeasts, we created Analyzing Yeasts by Reconstructing Ancestry of Homologs (AYbRAH), an open-source database of predicted and manually curated ortholog groups for 33 diverse fungi and yeasts in Dikarya, spanning 600 million years of evolution. OrthoMCL and OrthoDB were used to cluster protein sequence into ortholog and homolog groups, respectively; MAFFT and PhyML reconstructed the phylogeny of all homolog groups. Ortholog assignments for enzymes and small metabolite transporters were compared to their phylogenetic reconstruction, and curated to resolve any discrepancies. Information on homolog and ortholog groups can be viewed in the AYbRAH web portal (https://lmse.github.io/aybrah/), including functional annotations, predictions for mitochondrial localization and transmembrane domains, literature references and phylogenetic reconstructions. Ortholog assignments in AYbRAH were compared to HOGENOM, KEGG Orthology, OMA, eggNOG and PANTHER. PANTHER and OMA had the most congruent ortholog groups with AYbRAH, while the other phylogenomic databases had greater amounts of under-clustering, over-clustering or no ortholog annotations for proteins. Future plans are discussed for AYbRAH, and recommendations are made for other research communities seeking to create curated ortholog databases.

## Introduction

Yeasts are unicellular fungi that exploit diverse habitats on every continent, including the gut of wood boring beetles, insect frass, tree exudate, rotting wood, rotting cactus tissue, soil, brine solutions and fermenting juice (1). The most widely studied yeasts are true budding yeasts, which span roughly 400 million years of evolution in the subphylum Saccharomycotina (2), and possess a broad range of traits important to metabolic engineering. These include citrate and lipid accumulation in *Yarrowia* (3) and *Lipomyces* (4), thermotolerance in multiple lineages (5, 6), acid tolerance in *Pichia* (7) and *Zygosaccharomyces* (8), methanol utilization in *Komagataella* (9), osmotolerance in *Debaryomyces* (10), xylose to ethanol fermentation in multiple yeast lineages (11–13), alternative nuclear codon assignments (14), glucose and acetic acid co-consumption in *Zygosaccharomyces* (15) and aerobic ethanol production (the Crabtree effect) in multiple lineages (16–19). The complete genetic bases of these traits are mostly unknown, preventing their addition or removal in a chassis organism for biotechnology.

The distinction between orthologs, paralogs, ohnologs and xenologs plays an important role in bridging the genotype–phenotype gap across the tree of life (20). Briefly, orthologs are genes that arise from speciation and *typically* have a conserved function; paralogs and ohnologs emerge from locus and whole genome duplications, respectively, and *may* have a novel function; xenologs derive from horizontal gene transfer between organisms and do not necessarily have conserved function (21, 22). Knowledge of these types of genes has played an important role in deciphering *Saccharomyces cerevisiae*’s physiology. For example, the Adh2p paralog in *S. cerevisiae* consumes ethanol and evolved from an ancient Adh1p duplication whose kinetics favored ethanol production (23); the Saccharomycetaceae Whole Genome Duplication led to the *MPC2* and *MPC3* ohnologs in the *Saccharomyces* genus, which encode the fermentative and respirative subunits of the mitochondrial pyruvate carrier (24), respectively; the *URA1* xenolog from Lactobacillales enables uracil to be synthesized anaerobically in most Saccharomycetaceae yeasts (25). These examples demonstrate how understanding the origin of genes has narrowed the genotype–phenotype gap for fermentation in Saccharomycetaceae.

Many genomics studies have focused on the Saccharomycetaceae family, and to a lesser extent the CTG clade (26), but more can be learned about yeast metabolism by studying its evolution over a longer time horizon, especially with yeasts having deeper phylogeny (27). If we could study the metabolism of the mother of all budding yeasts, which we refer to as the Proto-Yeast, we could track the gains and losses of orthologs and function in all of her descendants to bridge various genotype–phenotype gaps. Proto-Yeast has evolved from her original state, making this direct study impossible, but we can reconstruct her metabolism through her living descendants. In recent yeasts, dozens of yeasts with deep phylogeny have been sequenced (28), paving the way for greater insight into the evolution of metabolism in yeasts beyond Saccharomycetaceae.

**Figure 1:**
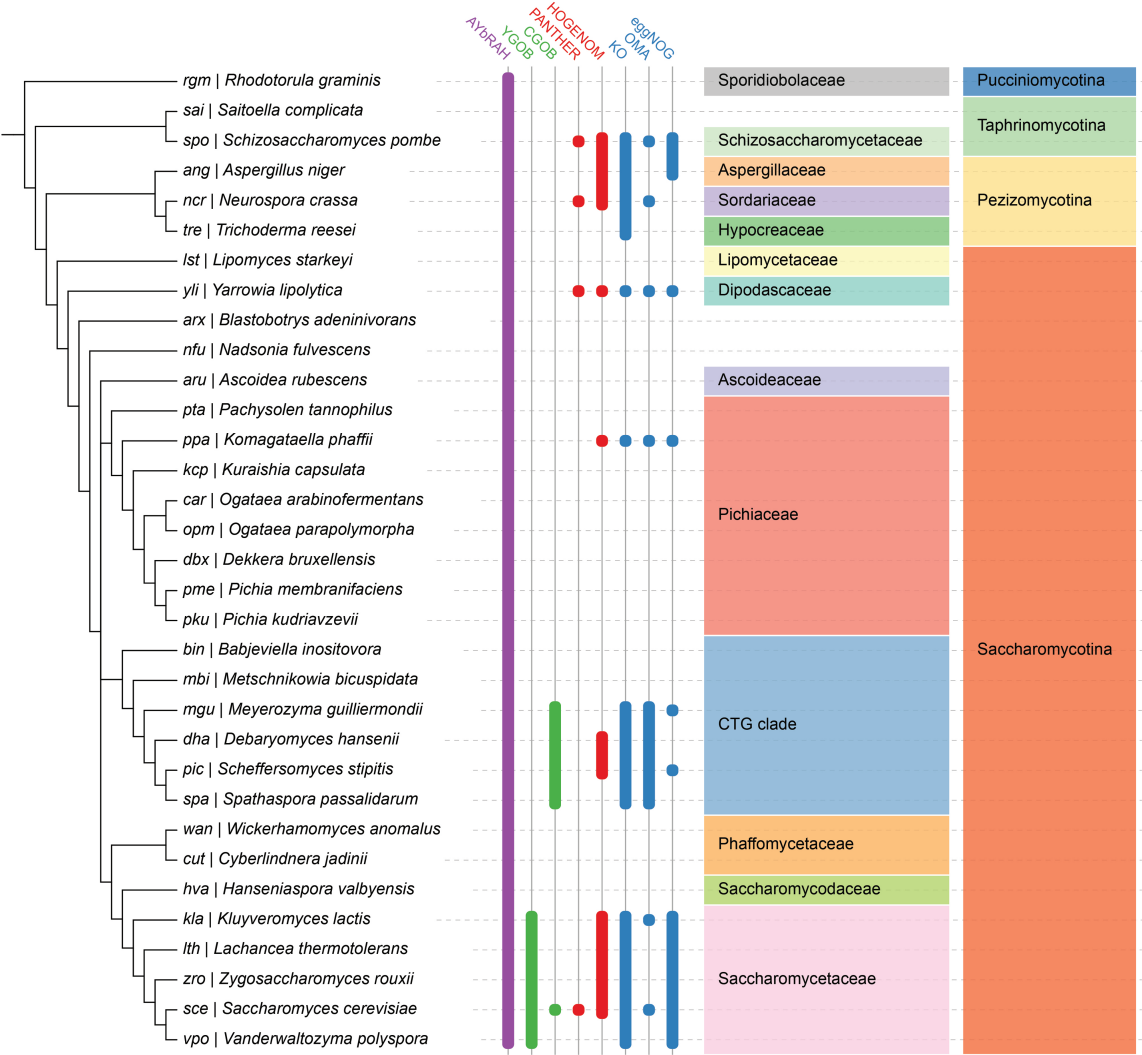
Ortholog database coverage for fungal and yeast genomes in AYbRAH, YGOB, CGOB, PANTHER, HOGENOM, KO, OMA and eggNOG. Ortholog assignments based on the manual curation of sequence similarity and synteny are shown in green columns, tree-based methods in red columns, graph-based methods in blue columns and a hybrid graph and tree-based method in the purple column. Many ortholog databases are well represented in Saccharomycetaceae and the CTG clade, which had their genomes sequenced during the 2000s (26). AYbRAH has ortholog assignments for species in Pichiaceae, Phaffomycetaceae and several *incertae sedis* families, which are not well represented in other ortholog databases, as these yeasts were recently sequenced (28). The well established phylogenomic databases span other yeast species not shown in this phylogeny, but they mostly belong to Saccharomycetaceae or the CTG clade.

**Figure 2:**
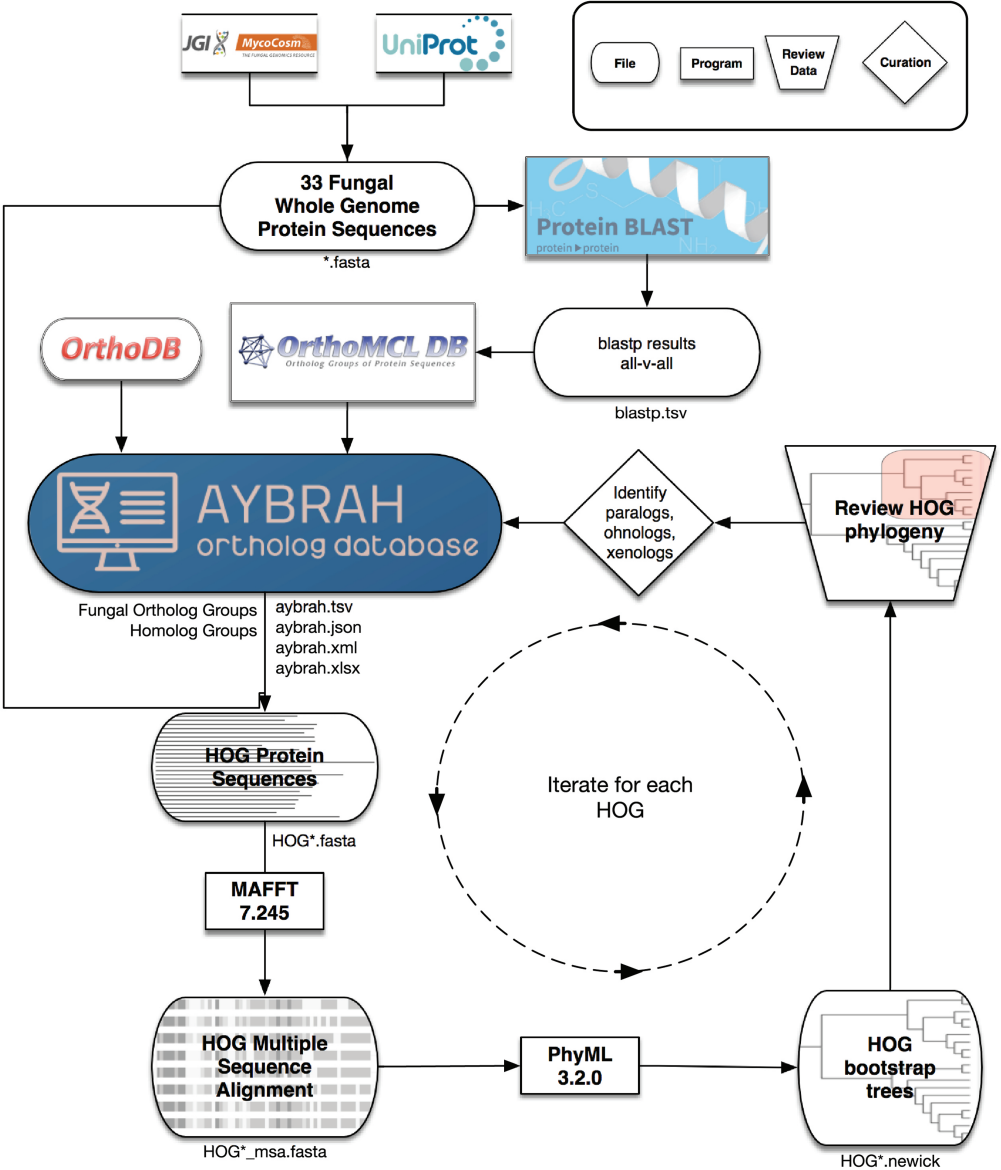
AYbRAH workflow for ortholog curation. A total of 33 fungal and yeast proteomes were downloaded from UniProt and MycoCosm. BLASTP computed the sequence similarity between all proteins. OrthoMCL clustered the proteins into putative Fungal Ortholog Groups (FOGs) using the BLASTP results. FOGs were clustered into HOmolog Groups (HOGs) using Fungi-level homolog assignments from OrthoDB. Multiple sequence alignments for each HOG were obtained with MAFFT, and 100 bootstrap phylogenetic trees were reconstructed with PhyML. The consensus phylogenetic trees for enzymes and transporters were reviewed and curated to differentiate between orthologs, paralogs, ohnologs and xenologs.

Ortholog databases are critical to facilitating comparative genomics studies and inferring protein function. Most of these databases are constructed using graph-based methods that rely on sequence similarity, while fewer databases use tree-based methods (29). Existing ortholog databases do not span diverse yeasts (Figure 1), and sometimes cannot distinguish between orthologs and paralogs (Tables S1 and S2). In addition to these databases, orthologs are identified on an *ad hoc* basis with OrthoMCL for comparative genomics studies (30, 31), or with the reciprocal best hit (RBH) method for genome-scale network reconstructions (GENREs) (32); these ortholog assignments often lack transparency or traceability, and therefore cannot be scrutinized or continuously improved by research communities. To solve these outlined problems, and ultimately improve our understanding of budding yeast physiology, we present Analyzing Yeasts by Reconstructing Ancestry of Homologs (AYbRAH; Figure 2). AYbRAH, derived from the Hebrew name Abra, mother of many, is an open-source database of predicted and manually curated orthologs, their function and their origin. The initial AYbRAH database was constructed using OrthoMCL and OrthoDB. PhyML was used to reconstruct the phylogeny of each homolog group. AYbRAH ortholog assignments for enzymes and small metabolite transporters were compared against their phylogenetic reconstruction and curated to resolve any discrepancies. We discuss the information available in the AYbRAH web portal (https://lmse.github.io/aybrah/), issues that arose from reviewing the accuracy of ortholog predictions, compare AYbRAH to established phylogenomic databases, discuss the benefits of open-source ortholog databases, future directions for AYbRAH, and offer recommendations to research communities looking to develop ortholog databases for other taxa.

**Table 1:** Fungal and yeast strain genomes in AYbRAH. Protein sequences were downloaded from UniProt or MycoCosm. Species were assigned to monophyletic or paraphyletic groups based on divergence time with *S. cerevisiae*

**Species**	**Strain**	**Group**	**Database**	**Reference**
*Rhodotorula graminis*	WP1	Saccharomycotina outgroup	MycoCosm	(73)
*Saitoella complicata*	NRRL Y-17804		MycoCosm	(28)
*Schizosaccharomyces pombe*	972h-		UniProt	(74)
*Aspergillus niger*	CBS 513.88		UniProt	(75)
*Neurospora crassa*	CBS708.71		UniProt	(76)
*Trichoderma reesei*	QM6a		UniProt	(77)
*Lipomyces starkeyi*	NRRL Y-11557	basal Saccharomycotina	MycoCosm	(28)
*Yarrowia lipolytica*	CLIB 122		UniProt	(78)
*Blastobotrys adeninivorans*	LS3		MycoCosm	(79)
*Nadsonia fulvescens var. elongata*	DSM 6959		MycoCosm	(28)
*Ascoidea rubescens*	NRRL Y17699		MycoCosm	(28)
*Pachysolen tannophilus*	NRRL Y-2460	Pichiaceae	MycoCosm	(28)
*Komagataella phaffii*	GS115		UniProt	(80)
*Kuraishia capsulata*	CBS 1993		UniProt	(81)
*Ogataea arabinofermentans*	NRRL YB-2248		MycoCosm	(28)
*Ogataea parapolymorpha*	NRRL Y-7560		UniProt	(83)
*Dekkera bruxellensis*	CBS 2499		MycoCosm	(82)
*Pichia membranifaciens*	NRRL Y-2026		MycoCosm	(28)
*Pichia kudriavzevii*	SD108		UniProt	(84)
*Babjeviella inositovora*	NRRL Y-12698	CTG clade	MycoCosm	(28)
*Metschnikowia bicuspidata*	NRRL YB-4993		MycoCosm	(28)
*Meyerozyma guilliermondii*	CBS 566		UniProt	(85)
*Debaryomyces hansenii*	CBS 767		UniProt	(78)
*Scheffersomyces stipitis*	CBS 6054		UniProt	(86)
*Spathaspora passalidarum*	NRRL Y-27907		UniProt	(30)
*Wickerhamomyces anomalus*	NRRL Y-366-8	Phaffomycetaceae & Saccharomycodaceae	MycoCosm	(28)
*Cyberlindnera jadinii*	NRRL Y-1542		MycoCosm	(28)
*Hanseniaspora valbyensis*	NRRL Y-1626		MycoCosm	(28)
*Kluyveromyces lactis*	CBS 2359	Saccharomycetaceae	UniProt	(78)
*Lachancea thermotolerans*	CBS 6340		UniProt	(87)
*Zygosaccharomyces rouxii*	CBS 732		UniProt	(87)
*Saccharomyces cerevisiae*	S288C		UniProt	(88)
*Vanderwaltozyma polyspora*	DSM 70294		UniProt	(89)

**Figure 3:**
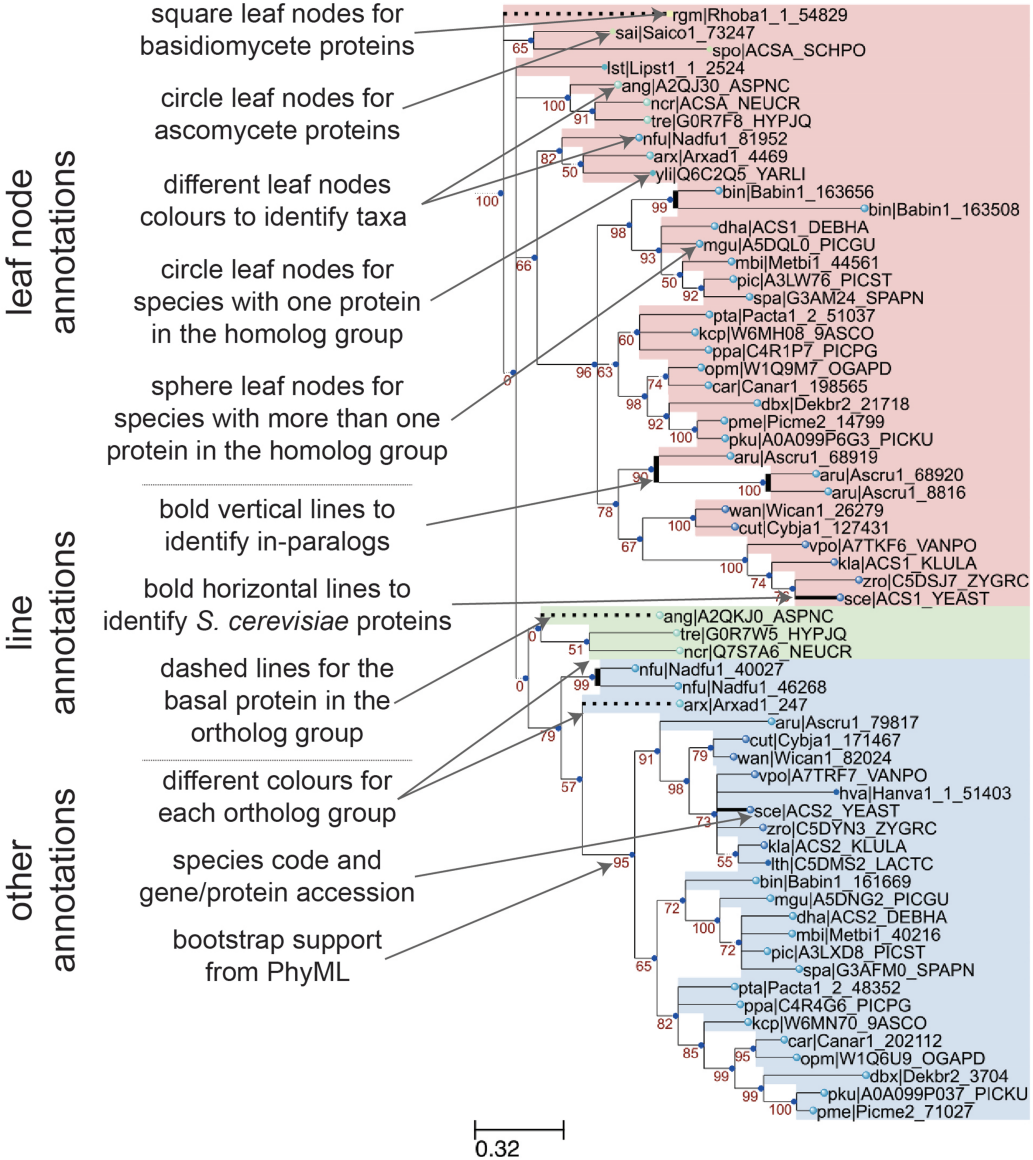
Annotation features of a sample phylogenetic tree in AYbRAH. Square and circle leaves indicate protein sequences in Basidiomycota or Ascomycota, respectively. Leaf nodes are colored based on taxonomic groups. Circle leaves are used for proteins with no paralogs in the same species, whereas sphere leaves are used to designate proteins with paralogs in the same species. Vertical bold lines indicate species-lineage expansions, which are sometimes called in-paralogs or co-orthologs (61). Horizontal bold lines designate *S. cerevisiae* proteins, which is the most widely studied eukaryote. Dashed lines indicate the most anciently diverged protein sequence in the ortholog group. Ortholog groups can be identified by color groups to help the visual inspection of ortholog assignments. The leaf names include a three-letter species code and a sequence accession. Internal nodes are labeled with the bootstrap values from phylogenetic reconstruction with PhyML.

**Figure 4:**
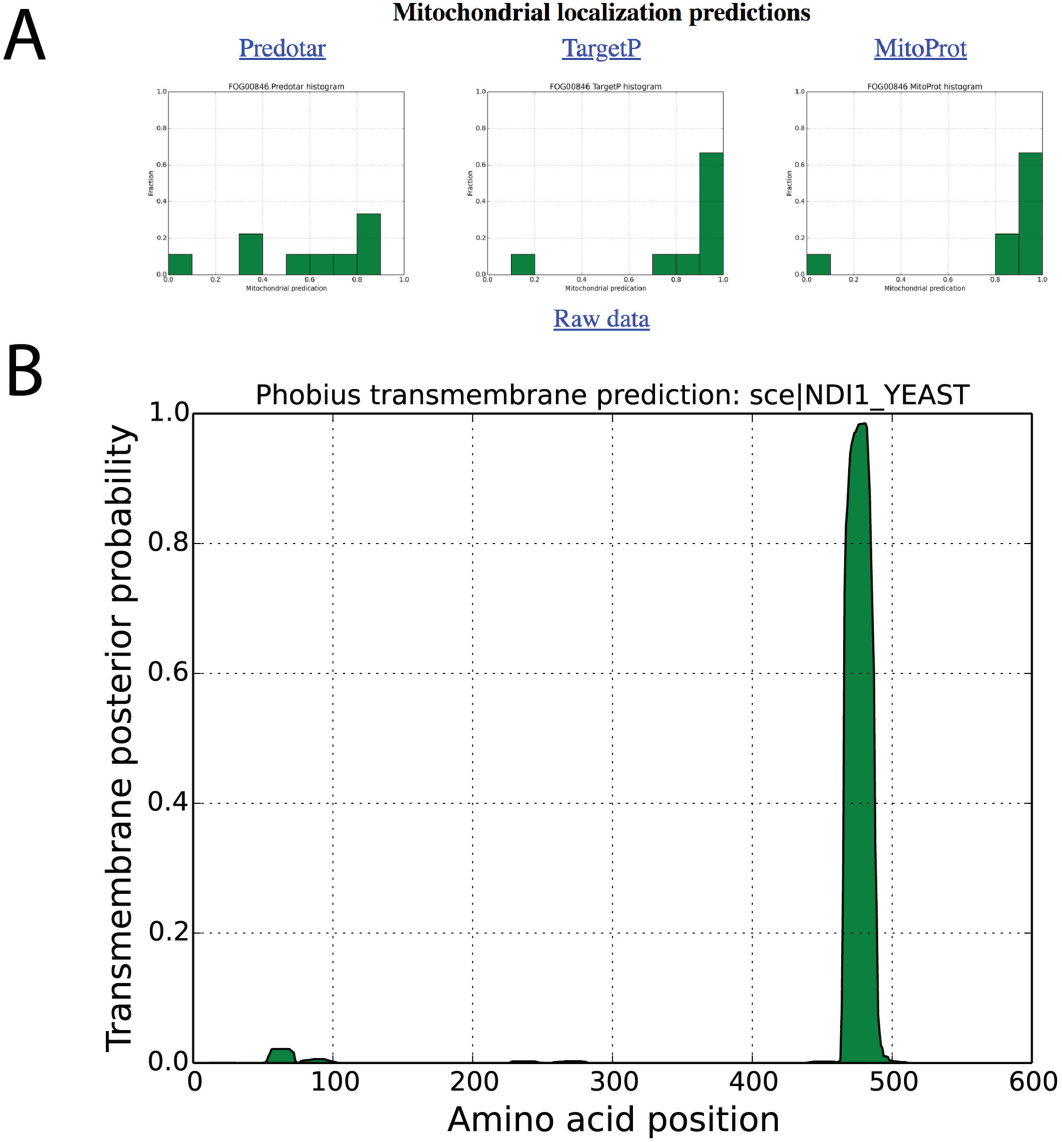
Localization predictions for internal NADH dehydrogenase (NDI1_YEAST) in AYbRAH. (A) Histogram plots are shown for mitochondrial localization predictions of Ndi1p orthologs Ndi1p predicted by Predotar, TargetP and MitoProt. (B) Transmembrane domain predictions computed for orthologous proteins by the Phobius web server.

## Methods

### Initial construction of AYbRAH

AYbRAH was created by combining several algorithms and databases in a pipeline (Figure 2). A total of 212 836 protein sequences from 33 organisms (Table 1) in Dikarya were downloaded from UniProt (33) and MycoCosm (34). OrthoMCL (35) clustered protein sequences into putative Fungal Ortholog Groups (FOGs); default parameters were used for BLASTP and OrthoMCL. The FOGs from OrthoMCL were coalesced into HOmolog Groups (HOGs) using Fungi-level homolog group assignments from OrthoDB v8 (36).

### AYbRAH curation

**Figure 5:**
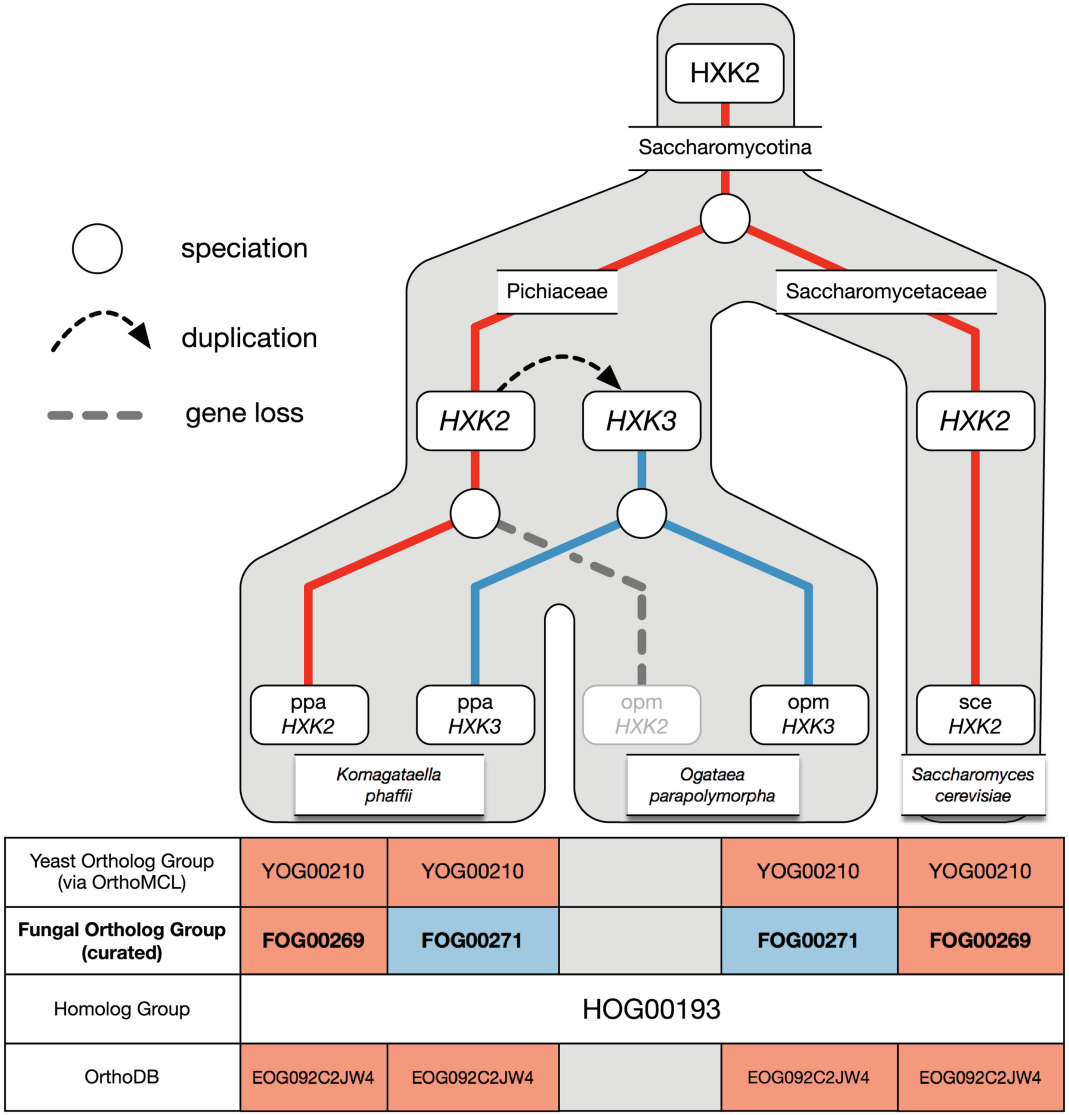
Example of over-clustering by OrthoMCL with the hexokinase family and its curation in AYbRAH. A gene duplication of *HXK2* in Pichiaceae led to the *HXK3* paralog. *HXK2* was subsequently lost in *Ogataea parapolymorpha* but maintained in *Komagataella phaffii*. OrthoMCL was unable to differentiate between the Hxk2p and Hxk3p orthologs. Both ortholog groups are also assigned to the same Fungi-level ortholog group in OrthoDB.

**Figure 6:**
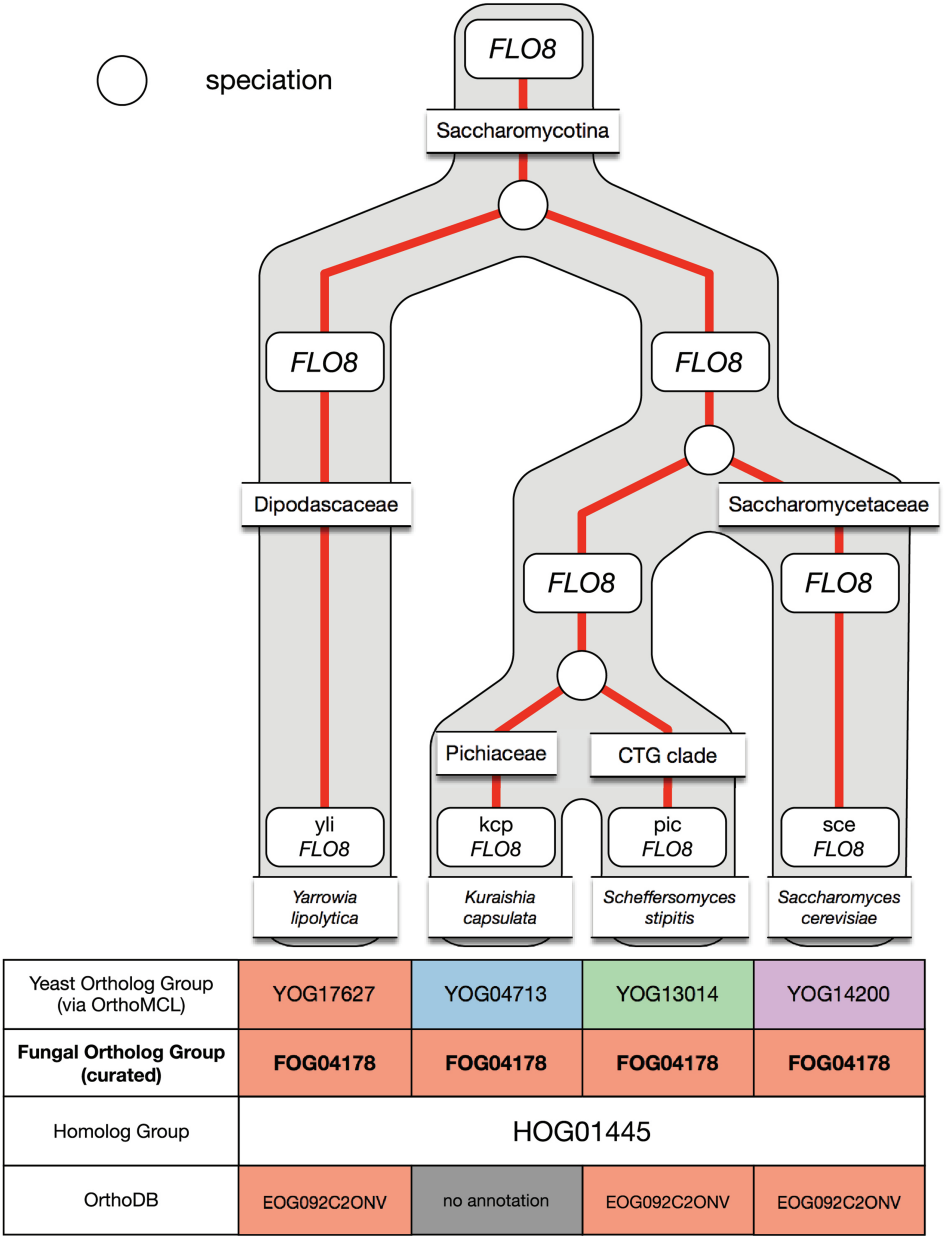
Example of under-clustering by OrthoMCL in the *FLO8* ortholog group and its curation in AYbRAH. OrthoMCL dispersed the Flo8p proteins into multiple ortholog groups due to the low sequence similarity between the proteins. The proteins were merged into one ortholog group.

Multiple sequence alignments were obtained for each HOG with MAFFT v7.245 (37) using a gap and extension penalty of 1.5. A total of 100 bootstrap trees were reconstructed for each HOG with PhyML v3.2.0 (38), optimized for tree topology and branch length. Consensus phylogenetic trees were generated for each HOG with SumTrees from DendroPy v4.1.0 (39), and trees were rendered with ETE v3 (40). The phylogenetic reconstruction for enzymes and metabolite transporters were reviewed when OrthoMCL failed to differentiate between orthologs and paralogs, caused by over-clustering (Figure 5), or when orthologous proteins were dispersed into multiple ortholog groups, caused by under-clustering (Figure 6). Orthologs were identified by visual inspection of the phylogenetic trees or with a custom ETE 3-based script (40).

### Annotating additional proteins

Additional steps were required to assign proteins to ortholog groups because OrthoMCL did not cluster all related proteins to ortholog groups, or because whole genome protein annotations were incomplete. First, proteins in OrthoDB homolog groups were added to new FOGs if they were not assigned to any FOG by OrthoMCL. Next, each organism had its genome nucleotide sequence queried by a protein sequence of the species closest relative for each FOG using TBLASTN (expect threshold of 1e-20). Annotated proteins were then queried against the TBLASTN hits to determine which proteins were annotated but not assigned to a FOG by OrthoMCL (misidentified) and which proteins were unannotated despite a match in its nucleotide sequence (unidentified). Proteins identified via TBLASTN with a sequence length <75% of the mean FOG sequence length were discarded from the candidate list. The remaining proteins were assigned to a HOG by its best hit via BLASTP, and to a FOG with pplacer (41) via the MAFFT add alignment option. The following examples highlight how misidentified and unidentified protein annotations were resolved in AYbRAH, respectively. First, Cybja1_169606 (A0A1E4RV95), which encodes NADP-dependent isocitrate dehydrogenase in *Cyberlindnera jadinii*, was not assigned to any ortholog group by OrthoMCL despite its high sequence similarity to other proteins. It was added to FOG00618 by pplacer (41) with a likelihood weight ratio of 1. Second, no 60S ribosomal protein L6 (FOG00006) was present in *Meyerozyma guilliermondii*’s protein annotation; it was identified by TBLASTN, annotated as mgu_AYbRAH_00173, and added to FOG00006 by pplacer with a 0.79 likelihood weight ratio (41).

### Comparison of ortholog groups

AYbRAH ortholog assignments were compared to OMA (42), PANTHER (43), HOGENOM (44), eggNOG (45) and KEGG Orthology (46). Phylogenomic annotations were downloaded from UniProt. Ortholog groups were assessed as congruent, over-clustered, under-clustered, over and under-clustered or no ortholog assignment relative to AYbRAH. AYbRAH ortholog groups were only compared with a database if an ortholog group in AYbRAH had proteins from species present in the other ortholog database. For example, FOG19691 consists of proteins from *Ascoidea rubescens*, *Pachysolen tannophilus*, *Kuraishia capsulata*, *Ogataea parapolymorpha*, *Dekkera bruxellensis*, *Pichia kudriavzevii*, *Pichia membranifaciens*, *Babjeviella inositovora*, *Wickerhamomyces anomalus* and *C. jadinii*. None of the phylogenomic databases have ortholog assignments for these organisms, and therefore cannot be compared with AYbRAH. Evolview v2 (47) was used to map ortholog databases coverage onto the yeast species tree.

### Subcellular localization prediction

Subcellular localization predictions for all proteins in the pan-genome were computed with MitoProt II (48), Predotar (49) and TargetP (50). The Phobius web server (51) was used to predict transmembrane domains for all proteins.

### Literature references

Literature references for characterized proteins were assigned to FOGs in AYbRAH. Additional references were obtained from paperBLAST (52), UniProt (33), *Saccharomyces* Genome Database (53), PomBase (54), *Candida* Genome Database (55) and *Aspergillus* Genome Database (56).

## AYbRAH overview

AYbRAH v0.1 and v0.2.3 database statistics are summarized in Table 2. In total, there are 214 498 protein sequences in the pan-genome for 33 yeasts and fungi; Pezizomycotina fungi were included in the database as an outgroup because they have genes that were present in Proto-Yeast’s ancestor, but subsequently lost. AYbRAH has 187 555 proteins (87% of the pan-proteome) that were assigned to 22 538 FOGs and 18 202 HOGs. Ortholog assignments are available in an Excel spreadsheet, a tab-separated file, orthoXML (57) and a JSON format.

**Table 2:** AYbRAH ortholog database statistics before and after curation. The initial ortholog assignments were obtained with OrthoMCL and OrthoDB. Additional proteins were annotated using TBLASTN. Ortholog groups for enzymes and small metabolite transporters were manually curated by visual inspection of homolog phylogeny and by identifying ortholog groups with an ETE 3 script (40). Ortholog groups were modified by adding unannotated proteins to existing groups via pplacer (41) or by collapsing multiple ortholog groups into a single ortholog group if there were no gene duplications in the homolog group (under-clustering)

	AYbRAH	
	v0.1	v0.2.3
Proteins	212 551	214 498
Proteins in AYbRAH	169 118 (79%)	187 555 (87%)
Fungal ortholog groups	14 249	22 538
Homolog groups	0	18 202
Manually curated ortholog groups	0	625
Electronically modified ortholog groups	0	3760

### The AYbRAH web portal

AYbRAH has a web page for each HOG with information on gene names, descriptions, gene origin (paralog, ohnolog and xenolog), literature references, localization predictions and phylogenetic reconstruction. A sample webpage for the acetyl-CoA synthetase can be seen in Supplementary Information. Protein families can be searched by FOG (FOG00404) or HOG (HOG00229) identification codes, gene names (*ACS1*), ordered locus (YAL054C), UniProt entry names (ACS1_YEAST) or protein accession codes from UniProt (Q01574), NCBI RefSeq (NP_009347.1) or EMBL (CAA47054.1).

A sample phylogenetic tree rendered by ETE v3 (40) and descriptions of its annotation features is shown in Figure 3 for the acetyl-CoA synthetase family (HOG00229). The initial ortholog assignments by OrthoMCL did not distinguish between the *ACS1* (FOG00404) and *ACS2* (FOG00405) paralogs. From this phylogeny, we can see that *ACS2* arose from a duplication from *ACS1*, because the basal species (*Rhodotorula graminis*, *Schizosaccharomyces pombe*, Pezizomycotina fungi) do not have *ACS2*, and the *ACS2* subtree has high bootstrap support (79%). Therefore, *ACS1* is the parent ortholog group to *ACS2*. This multi-level hierarchical relationship for ortholog groups was adopted in AYbRAH and was recently recommended by (58); current ortholog databases and Clusters of Orthologous Groups (COGs) collections treat these ortholog groups as equal or siblings. Discrepancies in ortholog assignments can be identified by comparing bootstrap support values for subtrees and ortholog assignments, as was done with *ACS1* and *ACS2*. Issues may be reported on GitHub or pull requests can be initiated for large changes to ortholog groups.

Snapshots for mitochondrial localization and transmembrane domain predictions are shown in Figures 4A and B for internal alternative NADH dehydrogenase, encoded by *NDI1* (FOG00846). Reviewing localization predictions for orthologous proteins with multiple algorithms enables researchers to make prudent decisions about protein localization, rather than relying on one method for one protein sequence. For example, Cybja1_131289 encodes internal alternative NADH dehydrogenase, yet its mitochondrial localization probability is 0.0019 with MitoProt II; all other mitochondrial predictions for Ndi1p orthologs are greater than 0.80 with MitoProt II. A review of the upstream nucleotide sequence of Cybja1_131289 indicates additional start codons that were not included in the protein annotation. MitoProt II predicts a mitochondrial localization probability of 0.5191 for the full protein sequence, which is more consistent with its orthologs.

## AYbRAH curation

OrthoMCL and OrthoDB are less computationally intensive than phylogenetic-based methods, but they are not always accurate (59). Curation was required to resolve incorrect ortholog assignments due to over-clustering and under-clustering.

### Over-clustering by OrthoMCL

Over-clustering has been described in past studies (60), which occurs when graph-based methods create ortholog groups that do not distinguish between orthologs and paralogs. Over-clustering by OrthoMCL was common in gene families with many duplications or high sequence similarities, such as the aldehyde dehydrogenase (HOG00216) and the major facilitator superfamily (HOG01031); adjusting parameters for BLASTP and OrthoMCL did not help differentiate between orthologs and paralogs in HOG00216 and neither did adding more proteomes to the OrthoMCL pipeline (results not shown). Figure 5 illustrates an example of over-clustering with a subset of the hexokinase family (HOG00193). In this phylogenetic reconstruction, one hexokinase gene was present in the ancestral yeast species, but a gene duplication in Pichiaceae led to the *HXK3* paralog; the *HXK2* ortholog is subsequently not maintained in *O. parapolymorpha*’s genome. OrthoMCL assigned the *HXK3* paralog to the same ortholog group as *HXK2*. The RBH method, commonly used for ortholog identification (62), would have also falsely identified *O. parapolymorpha*’s *HXK3* as orthologous to *S. cerevisiae*’s *HXK2*. This example highlights how the greediness of graph-based methods can misidentify orthologs, which has been shown for yeast ohnologs (59), and how incorrect ortholog assignments can be made with pairwise comparisons. Paralogs were identified from over-clustered ortholog groups by finding nodes with high bootstrap support in the consensus phylogenetic trees for homologs and migrating the proteins to new ortholog groups; in some cases orthologs were identified by reviewing the sequence alignment of homologs.

### Under-clustering by OrthoMCL

Under-clustering occurs when orthologous proteins are assigned to multiple ortholog groups. OrthoMCL was more prone to under-clustering for short protein sequences and proteins with low sequence similarity, such as subunits in the electron transport chain complexes and Flo8p. Figure 6 demonstrates under-clustering with a subset of the Flo8p family that was incorrectly assigned to multiple ortholog groups by OrthoMCL. Under-clustering was mostly resolved via a Python script that coalesced proteins into a new ortholog group when multiple FOGs were present in a HOG yet no organism had any gene duplications.

## Comparison of AYbRAH to other ortholog identification methods

### BLASTP scoring metrics

**Figure 7:**
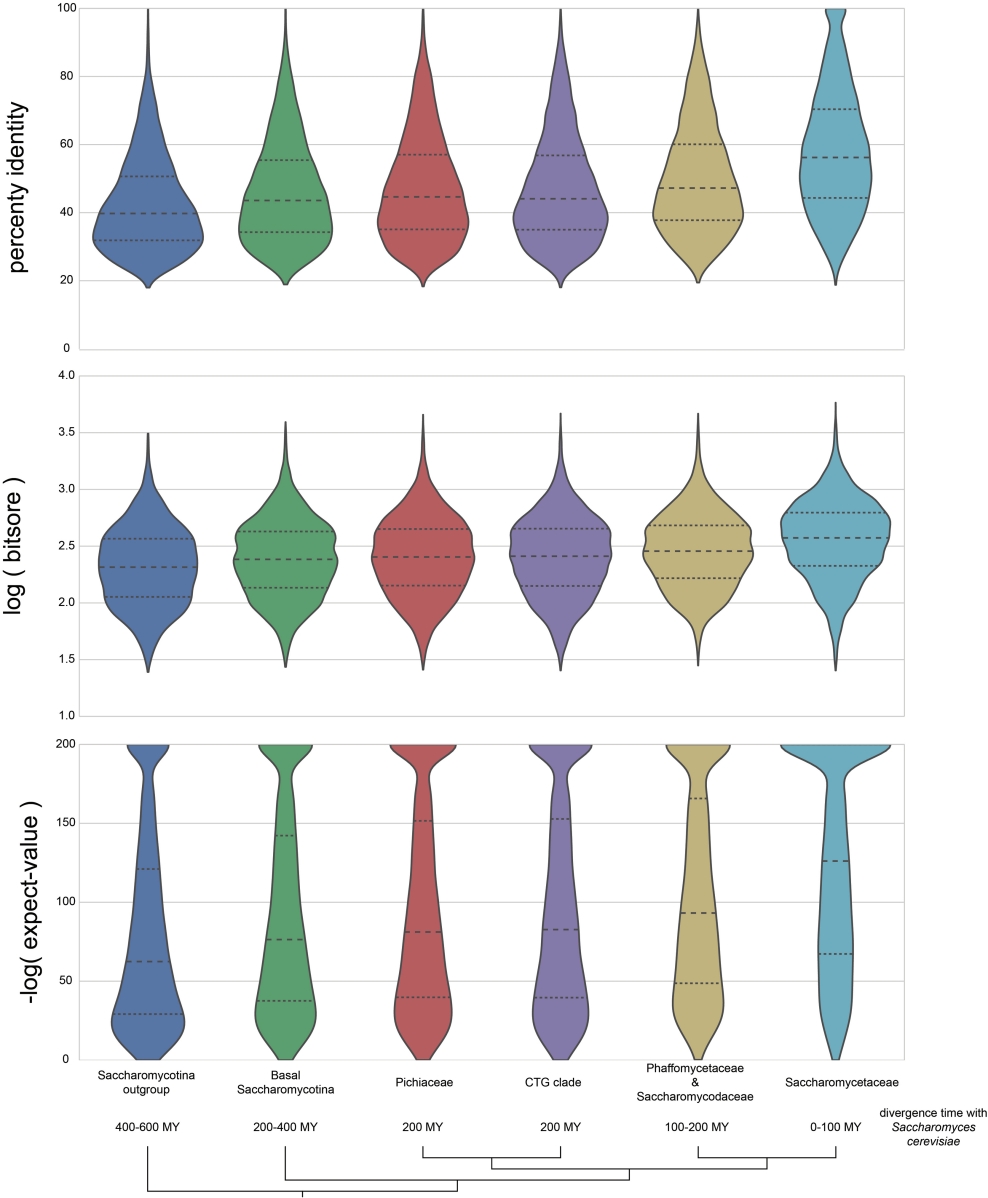
Distribution of BLASTP percent identities, logarithm of bit scores and negative logarithm of expect values for proteins orthologous to *S. cerevisiae*. The bottom half of orthologous proteins in the Saccharomycotina outgroup and Saccharomycetaceae has a percent identities of <40% and 58%, respectively; the bottom half of the expect-value ranges is >1e-60 and 1e-125 for the same groups. The wide and skewed distribution in the Saccharomycotina outgroup highlights the difficulty in making pairwise ortholog predictions for proteins with >400 millions of divergence in Dikarya fungi with BLASTP results; however, orthologs can be easily identified in the Saccharomycetaceae family because of their high sequence similarities and low expect values.

BLASTP is used as the basis for many ortholog predictions, including graph-based methods (29) and RBH (62). The distribution of percent identity, log(bit score) and −log(expect value) for proteins identified as orthologs to *S. cerevisiae* in AYbRAH are shown in Figure 7. Taxonomic groups include the Saccharomycotina outgroup, basal Saccharomycotina, Pichiaceae, CTG clade, Phaffomycetaceae and Saccharomycodaceae and Saccharomycetaceae (Table 1). The approximate divergence time with *S. cerevisiae* is 400–600 million years with the Saccharomycotina outgroup, 200–400 million years with the basal Saccharomycotina yeasts, 200 million years with Pichiaceae and CTG clades, 100–200 million years with Phaffomycetaceae and Saccharomycodaceae and 0–100 million years with Saccharomycetaceae. The distributions of percent identity, log(bit score), and -log(expect value) for proteins with 100–400 million years of divergence with *S. cerevisiae* are similar; however, the distributions skew differently for percent identity and -log(expect value) for the Saccharomycotina outgroup (400 million years of divergence) and Saccharomycetaceae (100 million years of divergence). Distributions for percent identity, log(bit score) and -log(expect value) for each species in AYbRAH are shown in Figures S1, S2 and S3. These results highlight the need to use phylogenetic methods and hidden Markov models to identify orthologs over long evolutionary timescales (43), but also enable orthologs to be identified by synteny and sequence similarity over smaller evolutionary time ranges (63, 64).

### Comparison of AYbRAH to well-established phylogenomic databases

Ortholog assignments in AYbRAH were compared with OMA, PANTHER, HOGENOM, eggNOG and KO (Table 3). OMA and PANTHER have the highest number of congruous ortholog groups with AYbRAH. Interestingly, PANTHER tends to over-cluster protein sequences into ortholog groups, while OMA tends to under-cluster. HOGENOM, eggNOG and KO have a high fraction of proteins not assigned to any ortholog groups, indicating that AYbRAH is able to identify more ortholog groups with OrthoMCL and OrthoDB.

**Table 3:** Comparison of ortholog assignments between AYbRAH and well-established phylogenomic databases. OMA and PANTHER are the most congruous with AYbRAH. Bold numbers indicate the greatest source of incongruency with AYbRAH. OMA and PANTHER are predicted to have more under-clustered and over-clustered groups relative to AYbRAH, respectively. HOGENOM, eggNOG and KO have a large number of proteins with no ortholog assignment

**Ortholog Database**	**FOGs compared**	**Congruent groups**	**Over-clustered groups**	**Under-clustered groups**	**Over and under-clustered groups**	**No ortholog group assignment**
OMA	8505	59%	5%	**19%**	3%	14%
PANTHER	7014	58%	**29%**	1%	4%	8%
HOGENOM	9393	50%	14%	11%	1%	**24%**
eggNOG	7827	48%	10%	4%	1%	**37%**
KO	9027	22%	16%	0%	0%	**62%**

Ten ortholog groups were randomly selected from the over-clustered groups in PANTHER and under-clustered groups in OMA to determine the source of the incongruency. It was found that 3 of the ten over-clustered ortholog groups in PANTHER were correctly annotated in AYbRAH, 1 ortholog group was correctly identified in PANTHER but under-clustered in AYbRAH, 1 ortholog group was not correctly identified in either database and 5 ortholog groups required further curation since the phylogenies are ambiguous. All ten ortholog groups from OMA were under-clustered, suggesting a systematic bias to not cluster proteins with lower sequence similarity; i.e., proteins identified as orthologous in AYbRAH were separated into two or more ortholog groups in OMA. Therefore, the PANTHER database is most closely aligned with AYbRAH. All other databases appear to be more prone to over-clustering or not have any annotation.

Orthology is inherently defined by phylogeny (65, 66). Clustering-based methods are well suited to cluster proteins into homolog groups, but it is not clear how these methods can properly identify orthologous proteins with one-dimensional sequence similarity alone, or identify xenologs without knowledge of a species tree. In our experience adding more diverse proteomes to OrthoMCL did not improve differentiation between orthologs and paralogs. PANTHER had a higher accuracy than other phylogenomic databases in our comparison with AYbRAH, despite PANTHER having fewer proteomes in its pan-genome. This is likely an outcome of its phylogenetic reconstruction of PANTHER families and its continued curation for two decades. Therefore, future methods should consider mapping new proteomes to existing databases, such as eggNOG-mapper (67) and TreeGrafter (68), rather than recomputing ortholog assignments, but also have a component of community curation.

## Applications of a curated ortholog database

Ortholog databases offer additional benefits beyond simply identifying orthologous proteins. These databases can be used to identify gene targets for functional characterization to functional genome annotation to streamlining GENRE; Galperin *et al*. (58) recently outlined some of the benefits and challenges to ortholog databases for microbial genomics. First, a curated ortholog database can serve as a repository for orthologs that have been screened and orthologs that require screening (69). Rather than characterizing all the orthologs in a handful of model organisms, research communities can broaden their efforts to understand the orthologs that do not exist in model organisms and the set of orthologs that do not have a conserved function with orthologs in model organisms. Second, a curated ortholog database can be used to improve and simplify genome annotation (69). Genes from newly sequenced organisms can be mapped to curated ortholog groups rather than using protein sequences from ortholog databases as queries in TBLASTN searches (70). New ortholog groups can be created for *de novo* genes or genes from recent duplications. Pulling annotations from a curated ortholog database has the advantage of unifying the names and descriptions of genes between organisms, as has been proposed for ribosomal subunits (71), and can reduce the number of genes that are misannotated or annotated as conserved hypothetical proteins. Finally, a curated ortholog database can be used to improve the quality and quantity of GENREs. GENREs inherently require a great deal of curation to identify orthologous proteins and their function, which is often not transparent. Refocusing this effort to curate ortholog groups and their function in open-source knowledgebase for pan-genomes can allow for improvements to be pushed to all GENREs, and for GENREs to be compiled for any taxonomic level, from kingdom to strain.

## Future plans for AYbRAH

### Integration with PANTHER

OrthoDB was chosen to cluster ortholog groups in AYbRAH into homolog groups because it spans more taxa than other phylogenomic databases and has ortholog assignments for different taxonomic ranks; however, it is less specific than PANTHER, despite the latter only having a few fungal proteome annotations. Future updates to AYbRAH will migrate the AYbRAH homolog group backbone from OrthoDB to PANTHER, and add the remaining fungi in PANTHER to increase its phylogenomic span. These include other fungal model organisms, fungi and yeasts having pathogenicity to humans or plants or fungi and yeasts occupying the following important taxonomic ranks: *Batrachochytrium dendrobatidis*, *Cryptococcus neoformans*, *Puccinia graminis*, *Ustilago maydis*, *Emericella nidulans*, *Neosartorya fumigata*, *Phaeosphaeria nodorum*, *Sclerotinia sclerotiorum*, *Candida albicans* and *Eremothecium gossypii*.

### Reconciling AYbRAH with YGOB and CGOB

The Yeast Gene Order Browser (YGOB) (63) and *Candida* Gene Order Browser (CGOB) (72) are the gold standard for ortholog databases in yeast genomics and were created using sequence similarity and synteny. YGOB and CGOB span roughly 112 and 239 million years of evolution, respectively, while AYbRAH spans 600 million years of evolution (2). Although AYbRAH has a broader pan-genomic coverage, YGOB and CGOB are expected to have better paralog and ohnolog assignments than AYbRAH because of its use of synteny. Future versions of AYbRAH will be reconciled with YGOB and CGOB.

### Coordinate-based protein annotations

It has been noted that genome protein annotations sometimes contain inaccuracies (72). For example, the protein translation Cybja1_131289 does not include its full N-terminal sequence. Another surprising shortfall of some genome annotations are genes that do not have any annotation. *Spathaspora passildarium*’s genome encodes have two *PHO3* homologs in tandem, but only one protein is currently annotated. AYbRAH will adopt the genomic coordinate-based system used in YGOB and CGOB (72) to improve protein annotations.

## Conclusion

In conclusion, we developed AYbRAH as an open-source ortholog database for yeasts and fungi because existing phylogenomic databases do not span diverse yeasts and sometimes cannot distinguish between orthologs, paralogs and xenologs. Manual curation was required for gene families with high sequence similarity, often arising from recent gene duplications, and with gene families with low sequence similarity. Curated ortholog databases can be implemented for other taxa to improve their genome annotations using PANTHER and other tree-based methods.

## Abbreviations

(AYbRAH) Analyzing Yeasts by Reconstructing Ancestry of Homologs; (CGOB) *Candida* Gene Order Browser; (COG) Clusters of Orthologous Groups; (FOG) Fungal Ortholog Group; (GENRE) Genome-scale Network REconstruction; (HOG) Homolog Group; (YGOB) Yeast Gene Order Browser; (RBH) Reciprocal Best Hit

## Availability of data

AYbRAH database files and additional files, such as phylogenetic trees and sequence alignments, can be found at https://github.com/LMSE/aybrah.

## Supplementary Material

aybrah_SI_baz022

supp_fig1_oid_by_pid_baz022

supp_fig2_oid_score_log10_baz022

supp_fig3_oid_evalue_neglog10_baz022

## Notes

**Database URL: https://lmse.github.io/aybrah/**
