# Supplementary material for: AYbRAH: a curated ortholog database for yeasts and fungi spanning 600 million years of evolution
Source: Database (Oxford). 2019 Mar 20;2019:baz022. doi: 10.1093/database/baz022 (PMC6425859; doi:10.1093/database/baz022)
Supplement: aybrah_SI_baz022 [file aybrah_si_baz022.pdf]

Supplemental Materials: AYbRAH: a curated ortholog database for yeasts and fungi spanning 600 million years of evolution

October 8, 2018

Contents

1 Comparison of AYbRAH orthology assignments to published ortholog databases 2

2 Sample webpages for homolog groups 6

1 Comparison of AYbRAH orthology assignments to published ortholog databases

Table S1: **Comparison of manually curated acetyl-Coenzyme A synthetase orthologs in AYbRAH to highly cited ortholog databases.** N/A indicates genomes that do not have orthology relationships in the public database but have been assigned orthology with AYbRAH. Omission indicates genomes that have annotations in the public ortholog database but do not have an annotation for the given gene. PANTHER is the only database that can distinguish between the three ACS ortholog groups; ACS3 is assigned to a different PANTHER family despite the shared ancestry of all the orthologs. KEGG can only differentiate between ACS1 and ACS3 ortholog groups, while all other database orthologly assignments are polyphyletic. EggNOG includes FOG07524 and FOG07525 in the ACS ortholog group, which both have predicted acetoacetate-CoA ligase activity.

AYbRAH Annotation		Ortholog DB	spo	ang	ncr	yli	ppa	pic	kla	sce
ACS1	FOG00404	PANTHER	PTHR24095:SF14	N/A	PTHR24095:SF14	PTHR24095:SF14	N/A	N/A	N/A	PTHR24095:SF14
ACS2	FOG00405						N/A	N/A	N/A	PTHR24095:SF245
ACS3	FOG00406			N/A	PTHR43347:SF3					
ACS1	FOG00404	KEGG	K01895	K01895	K01895	K01895	K01895	K01895	K01895	K01895
ACS2	FOG00405						K01895	K01895	K01895	K01895
ACS3	FOG00406			K01908	K01908					
ACS1	FOG00404	OrthoMCL	OG5_126680	N/A	OG5_126680	OG5_126680	N/A	OG5_126680	OG5_126680	OG5_126680
ACS2	FOG00405						N/A	OG5_126680	OG5_126680	OG5_126680
ACS3	FOG00406			N/A	OG5_126680					
ACS1	FOG00404	OrthoDB	EOG8HQC0H	EOG8HQC0H	EOG8HQC0H	EOG8HQC0H	EOG8HQC0H	EOG8HQC0H	EOG8HQC0H	EOG8HQC0H
ACS2	FOG00405						EOG8HQC0H	EOG8HQC0H	EOG8HQC0H	EOG8HQC0H
ACS3	FOG00406			omission	EOG8HQC0H					
ACS1	FOG00404	EggNOG	KOG1175	KOG1175	KOG1175	KOG1175	KOG1175	KOG1175	KOG1175	KOG1175
ACS2	FOG00405						KOG1175	KOG1175	KOG1175	KOG1175
ACS3	FOG00406			KOG1175	KOG1175					
HYP	FOG07524			KOG1175	KOG1175					
HYP	FOG07525			KOG1175	KOG1175					

Table S2: **Comparison of manually curated Type II NADH dehydrogenase (NDH2) orthologs in AYbRAH to highly cited ortholog databases.** N/A indicates genomes that do not have orthology relationships in the public database but have been assigned orthology with AYbRAH. Omission indicates genomes that have annotations in the public ortholog database but do not have an annotation for the given gene. PANTHER is able to distinguish between most orthologs in the NDH2 family, with the exception of NDE1 and NDE2; NDE0 is in a different PANTHER family than the rest of the NDH2 genes. KEGG is the only other database that can differentiate between some NDH2 genes; the genes are split between the older NDI0/NDE0 ortholog group and more recent NDE1/NDI1 ortholog group. PANTHER and EggNOG contain additional genes not included in other ortholog databases, which may represent ancient paralogs having lower sequence similarities than other NDH2 paralogs. AIF1, which can localize to the mitochondria in S. cerevisiae, is in the same subfamily as NDE0 in PANTHER; the other inconsistency is an Aspergillus niger gene (FOG07265) paralogous to a characterized external NADH dehydrogenase in Neurospora crassa (FOG07264) in EggNOG (Carneiro et al., 2007).

AYbRAH annotation		Ortholog Database	spo	ang	ncr	yli	ppa	pic	kla	sce
NDI0	FOG00837	PANTHER		N/A	PTHR43706:SF11					
NDE0	FOG00838			N/A	PTHR42913:SF2	PTHR42913:SF2	N/A		N/A	
AIF1	FOG11982						N/A			PTHR42913:SF2
NDE1	FOG00839		PTHR43706:SF1	N/A	PTHR43706:SF1	PTHR43706:SF1	N/A	N/A	N/A	PTHR43706:SF1
NDE2	FOG00845									PTHR43706:SF1
NDI1	FOG00846								N/A	PTHR43706:SF10
NDI0	FOG00837	KEGG		omission	K03885					
NDE0	FOG00845			omission	K03885	K03885	K03885		K03885	
NDE1	FOG00845		K17871	K17871	omission	K17871	K17871	K17871	K17871	K17871
NDE2	FOG00845									K17871
NDI1	FOG00845								K17871	K17871
NDI0	FOG00837	OrthoMCL		N/A	OG5_126960					
NDE0	FOG00838			N/A	OG5_126960	OG5_126960	N/A		OG5_126960	
NDE1	FOG00839		OG5_126960	N/A	OG5_126960	OG5_126960	N/A	OG5_126960	OG5_126960	OG5_126960
NDE2	FOG00845									OG5_126960
NDI1	FOG00846								OG5_126960	OG5_126960
NDI0	FOG00837	OrthoDB		EOG8P8D18	EOG8P8D18					
NDE0	FOG00838			EOG8P8D18	EOG8P8D18	EOG8P8D18	EOG8P8D18		EOG8P8D18	
NDE1	FOG00839		EOG8P8D18	EOG8P8D18	EOG8P8D18	EOG8P8D18	EOG8P8D18	EOG8P8D18	EOG8P8D18	EOG8P8D18
NDE2	FOG00845									EOG8P8D18
NDI1	FOG00846								EOG8P8D18	EOG8P8D18
NDI0	FOG00837	EggNOG		KOG2495	KOG2495					
NDE0	FOG00838			KOG2495	KOG2495	KOG2495	KOG2495		KOG2495	
NDE1	FOG00839		KOG2495	KOG2495	KOG2495	KOG2495	KOG2495	KOG2495	KOG2495	KOG2495
NDE2	FOG00845									KOG2495
NDI1	FOG00846								KOG2495	KOG2495
HYP	FOG07265			KOG2495						

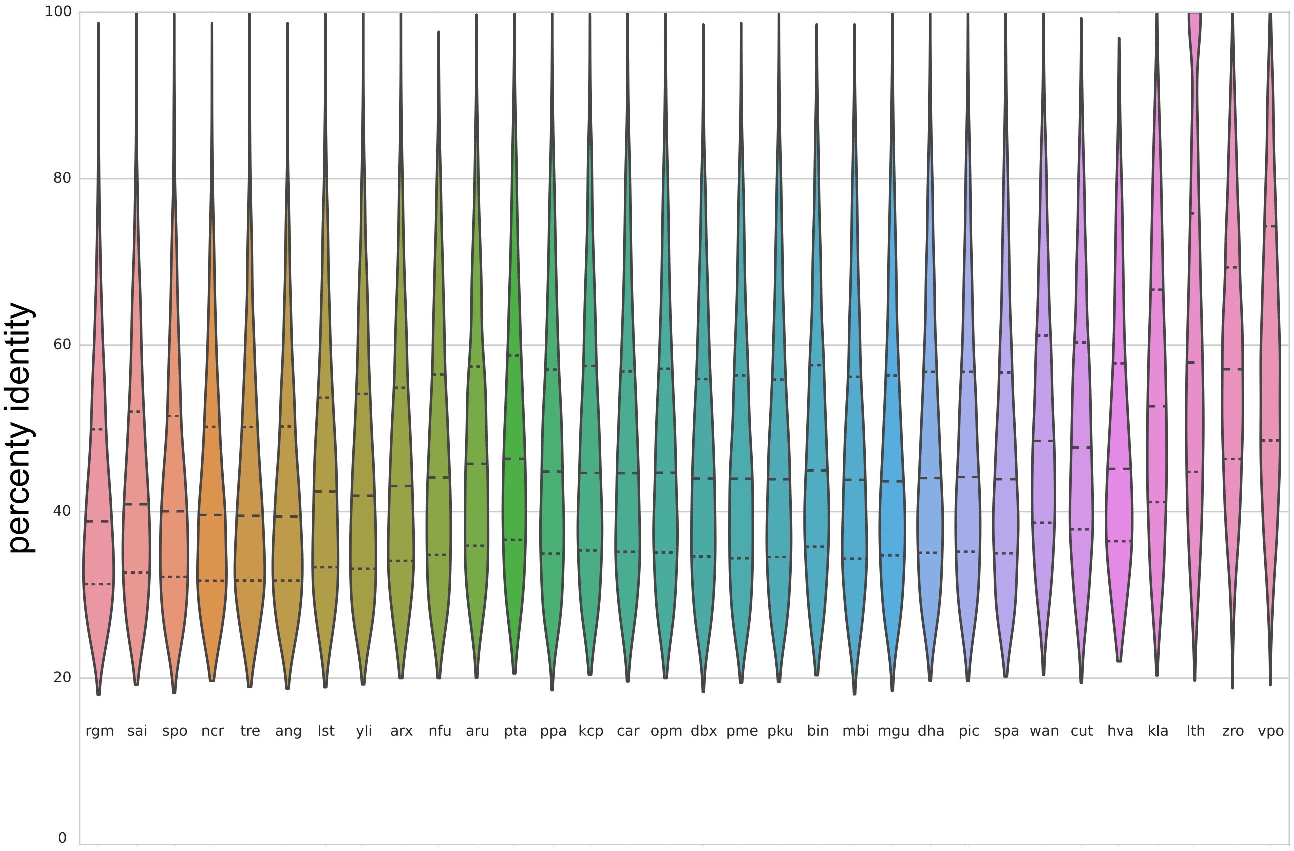

Figure S1: Distributions of BLASTP percent identities for proteins identified as orthologous to *Saccharomyces cerevisiae* in AYbRAH.

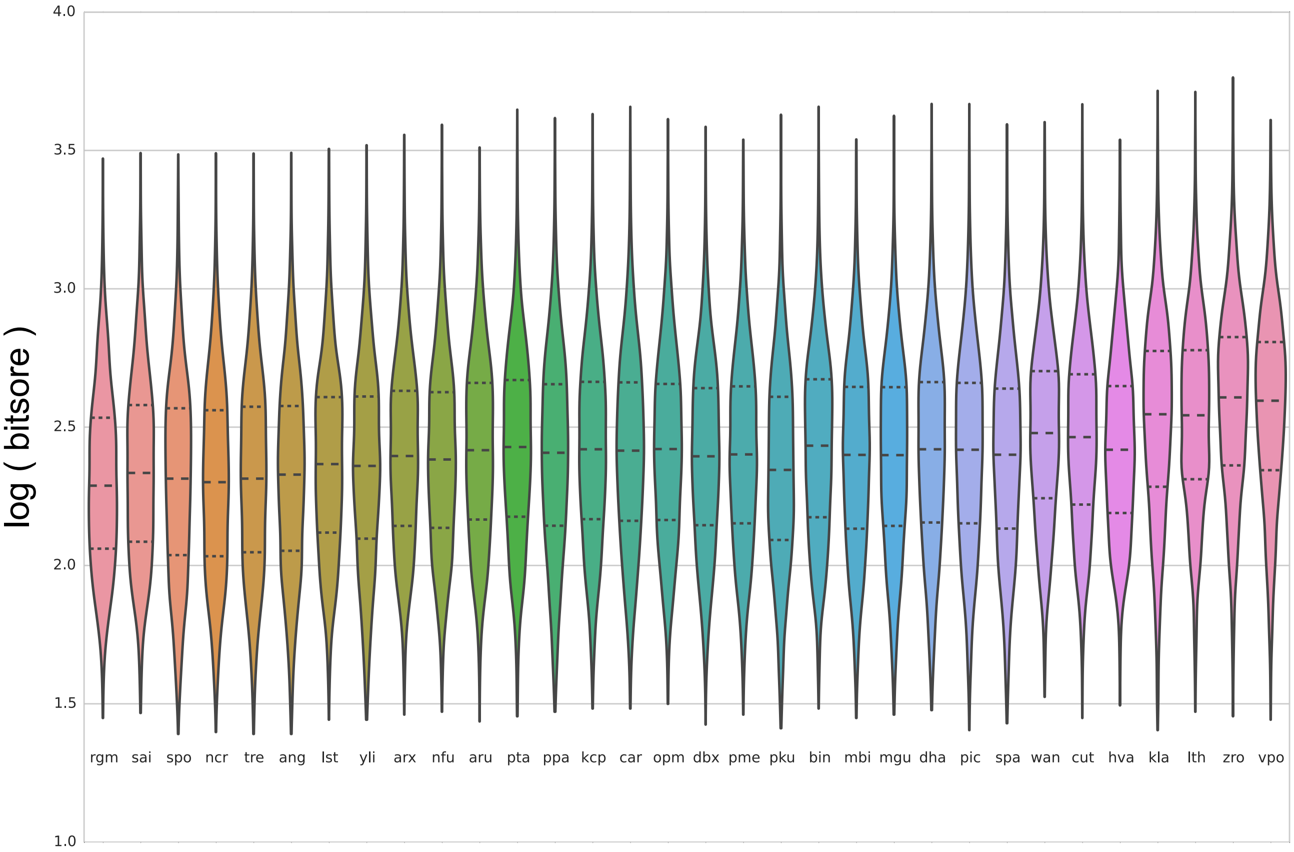

Figure S2: Distributions of logarithm BLASTP bitscores for proteins orthologous to *Saccharomyces cerevisiae* in AYbRAH.

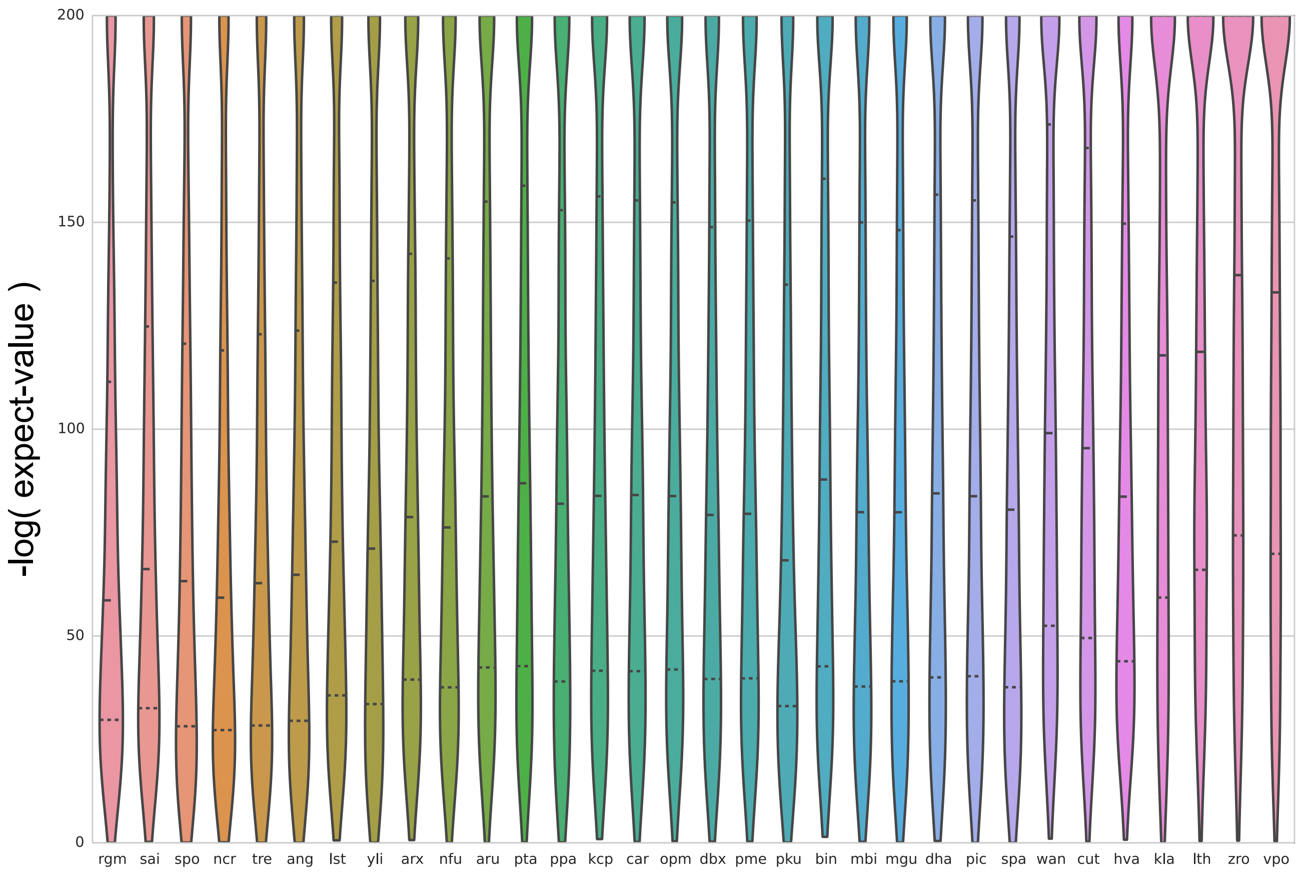

Figure S3: Distributions of negative logarithm of BLASTP expect-values for proteins orthologous to *Saccharomyces cerevisiae* in AYbRAH.

2 Sample webpages for homolog groups

Home

HOG00227 HAD-like hydrolase superfamily. DOG/GPP family
HOG00229
HOG00230 acetyl-CoA hydrolase/transferase family

FASTA
MAFFT sequence alignment
Phyml trees
Gblocks
Phobius predictions

FOG00404
EOG8HQC0H
ACS1
sce:ACS1

Genes: 34

Protein description
Acetyl-coA synthetase isoform expressed with non-fermentable carbon sources. Spo gene expressed with fermentable carbon sources.

SGD Description
Acetyl-coA synthetase isoform; along with Acs2p, acetyl-coA synthetase isoform is the nuclear source of acetyl-coA for histone acetylation; expressed during growth on nonfermentable carbon sources and under aerobic conditions

PomBase Description
acetyl-CoA ligase (predicted)

AspGD Description
Putative acetyl-CoA synthase

References

Armitt S, *et al*. (1976 Feb). Analysis of acetate non-utilizing (acu) mutants in Aspergillus nidulans.

Payton M, *et al*. (1976 May). Agar as a carbon source and its effect on the utilization of other carbon sources by acetate non-utilizing (acu) mutants of Aspergillus nidulans.

Frenkel EP, *et al*. (1977 Jan 25). Purification and properties of acetyl coenzyme A synthetase from bakers' yeast.

Hynes MJ, *et al*. (1977 Sep). Induction of the acetamidase of Aspergillus nidulans by acetate metabolism.

Midelfort CF, *et al*. (1978 Oct 25). The stereochemical course of acetate activation by yeast acetyl-CoA synthetase.

Bal J, *et al.* (1979 Dec). Allele specific and locus non-specific suppressors in Aspergillus nidulans.

Kelly JM, *et al.* (1981 Apr). The regulation of phosphoenolpyruvate carboxykinase and the NADP-linked malic enzyme in Aspergillus nidulans.

Sandeman RA, *et al.* (1989 Jul). Isolation of the facA (acetyl-coenzyme A synthetase) and acuE (malate synthase) genes of Aspergillus nidulans.

Connerton IF, *et al.* (1990 Mar). Comparison and cross-species expression of the acetyl-CoA synthetase genes of the Ascomycete fungi, Aspergillus nidulans and Neurospora crassa.

Lloyd AT, *et al.* (1991 Nov). Codon usage in Aspergillus nidulans.

Sandeman RA, *et al.* (1991 Sep). Molecular organisation of the malate synthase genes of Aspergillus nidulans and Neurospora crassa.

Birch PR, *et al.* (1992). Nucleotide sequence of a gene from Phanerochaete chrysosporium that shows homology to the facA gene of Aspergillus nidulans.

Maconochie MK, *et al.* (1992 Aug). The acu-1 gene of Coprinus cinereus is a regulatory gene required for induction of acetate utilisation enzymes.

De Virgilio C, *et al.* (1992 Dec). Cloning and disruption of a gene required for growth on acetate but not on ethanol: the acetyl-coenzyme A synthetase gene of Saccharomyces cerevisiae.

Kujau M, *et al.* (1992 Mar). Characterization of mutants of the yeast Yarrowia lipolytica defective in acetyl-coenzyme A synthetase.

Saleeba JA, *et al.* (1992 Nov). Characterization of the amdA-regulated aciA gene of Aspergillus nidulans.

Steensma HY, *et al.* (1993 Apr). Genetic and physical localization of the acetyl-coenzyme A synthetase gene ACS1 on chromosome I of Saccharomyces cerevisiae.

Martínez-Blanco H, *et al.* (1993 Aug 25). Characterisation of the gene encoding acetyl-CoA synthetase in Penicillium chrysogenum: conservation of intron position in plectomycetes.

Gouka RJ, *et al.* (1993 Jan). Development of a new transformant selection system for Penicillium chrysogenum: isolation and characterization of the P. chrysogenum acetyl-coenzyme A synthetase gene (facA) and its use as a homologous selection marker.

Sealy-Lewis HM, *et al.* (1994 Jan). A new selection method for isolating mutants defective in acetate utilisation in Aspergillus nidulans.

Van den Berg MA, *et al.* (1995 Aug 1). ACS2, a Saccharomyces cerevisiae gene encoding acetyl-coenzyme A synthetase, essential for growth on glucose.

Kratzer S, *et al.* (1995 Aug 8). Carbon source-dependent regulation of the acetyl-coenzyme A synthetase-encoding gene ACS1 from Saccharomyces cerevisiae.

van den Berg MA, *et al.* (1996 Nov 15). The two acetyl-coenzyme A synthetases of Saccharomyces cerevisiae differ with respect to kinetic properties and transcriptional regulation.

de Jong-Gubbels P, *et al.* (1997 Aug 1). The Saccharomyces cerevisiae acetyl-coenzyme A synthetase encoded by the ACS1 gene, but not the ACS2-encoded enzyme, is subject to

glucose catabolite inactivation.

Clutterbuck AJ, *et al*. (1997 Jun). The validity of the Aspergillus nidulans linkage map.

Todd RB, *et al*. (1998 Apr 1). FacB, the Aspergillus nidulans activator of acetate utilization genes, binds dissimilar DNA sequences.

Stemple CJ, *et al*. (1998 Dec). The facC gene of Aspergillus nidulans encodes an acetate-inducible carnitine acetyltransferase.

Papadopoulou S, *et al*. (1999 Sep 1). The Aspergillus niger acuA and acuB genes correspond to the facA and facB genes in Aspergillus nidulans.

Dessen P, *et al*. (2000 Feb 22). The PAUSE software for analysis of translational control over protein targeting: application to E. nidulans membrane proteins.

Brock M, *et al*. (2000 Mar). Methylcitrate synthase from Aspergillus nidulans: implications for propionate as an antifungal agent.

Jones IG, *et al*. (2001 Feb). ADHII in Aspergillus nidulans is induced by carbon starvation stress.

Lodi T, *et al*. (2001 Sep). Three target genes for the transcriptional activator Cat8p of Kluyveromyces lactis: acetyl coenzyme A synthetase genes KlACS1 and KlACS2 and lactate permease gene KlJEN1.

Hynes MJ, *et al*. (2002 Jan). Regulation of the acuF gene, encoding phosphoenolpyruvate carboxykinase in the filamentous fungus Aspergillus nidulans.

Kumar A, *et al*. (2002 Mar 15). Subcellular localization of the yeast proteome.

Flipphi M, *et al*. (2002 May 15). Characteristics of physiological inducers of the ethanol utilization (alc) pathway in Aspergillus nidulans.

Flipphi M, *et al*. (2003 Apr 4). Onset of carbon catabolite repression in Aspergillus nidulans. Parallel involvement of hexokinase and glucokinase in sugar signaling.

Zeeman AM, *et al*. (2003 Jan 15). The acetyl co-enzyme A synthetase genes of Kluyveromyces lactis.

Sickmann A, *et al*. (2003 Nov 11). The proteome of Saccharomyces cerevisiae mitochondria.

Flipphi M, *et al*. (2003 Sep). Relationships between the ethanol utilization (alc) pathway and unrelated catabolic pathways in Aspergillus nidulans.

Brock M, *et al*. (2004 Aug). On the mechanism of action of the antifungal agent propionate.

Sims AH, *et al*. (2004 Feb). Use of expressed sequence tag analysis and cDNA microarrays of the filamentous fungus Aspergillus nidulans.

Jogl G, *et al*. (2004 Feb 17). Crystal structure of yeast acetyl-coenzyme A synthetase in complex with AMP.

Takasaki K, *et al*. (2004 Mar 26). Fungal ammonia fermentation, a novel metabolic mechanism that couples the dissimilatory and assimilatory pathways of both nitrate and ethanol. Role of acetyl CoA synthetase in anaerobic ATP synthesis.

Zhang YQ, *et al*. (2004 Oct). Connection of propionyl-CoA metabolism to polyketide biosynthesis in Aspergillus nidulans.

David H, *et al*. (2006). Metabolic network driven analysis of genome-wide transcription data from Aspergillus nidulans.

Mogensen J, *et al*. (2006 Aug). Transcription analysis using high-density micro-arrays of Aspergillus nidulans wild-type and creA mutant during growth on glucose or ethanol.

Takahashi H, *et al*. (2006 Jul 21). Nucleocytosolic acetyl-coenzyme a synthetase is required for histone acetylation and global transcription.

Hynes MJ, *et al*. (2006 May). Regulatory genes controlling fatty acid catabolism and peroxisomal functions in the filamentous fungus Aspergillus nidulans.

Salazar M, *et al*. (2009 Dec). Uncovering transcriptional regulation of glycerol metabolism in Aspergilli through genome-wide gene expression data analysis.

Shimizu M, *et al*. (2009 Jan). Proteomic analysis of Aspergillus nidulans cultured under hypoxic conditions.

Flipphi M, *et al*. (2009 Mar). Biodiversity and evolution of primary carbon metabolism in Aspergillus nidulans and other Aspergillus spp.

Hynes MJ, *et al*. (2010 Jul). ATP-citrate lyase is required for production of cytosolic acetyl coenzyme A and development in Aspergillus nidulans.

Oh YT, *et al*. (2010 Mar). Proteomic analysis of early phase of conidia germination in Aspergillus nidulans.

Wendland J, *et al*. (2011 Dec). Genome evolution in the eremothecium clade of the Saccharomyces complex revealed by comparative genomics.

Saykhedkar S, *et al*. (2012 Jul 26). A time course analysis of the extracellular proteome of Aspergillus nidulans growing on sorghum stover.

Georgakopoulos P, *et al*. (2012 Nov). SAGA complex components and acetate repression in Aspergillus nidulans.

Nakamura T, *et al*. (2012 Sep). Impaired coenzyme A synthesis in fission yeast causes defective mitosis, quiescence-exit failure, histone hypoacetylation and fragile DNA.

Carpy A, *et al*. (2014 Aug). Absolute proteome and phosphoproteome dynamics during the cell cycle of Schizosaccharomyces pombe (Fission Yeast).

Beckley JR, *et al*. (2015 Dec). A Degenerate Cohort of Yeast Membrane Trafficking DUBs Mediates Cell Polarity and Survival.

Malecki M, *et al*. (2016 Nov 25). Functional and regulatory profiling of energy metabolism in fission yeast.

Mitochondrial localization predications

Predotar	TargetP	MitoProt

Mitochondrial localization predications

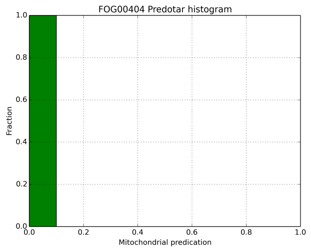

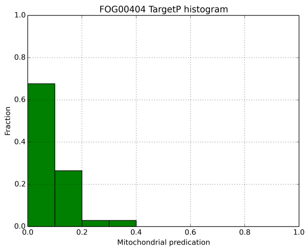

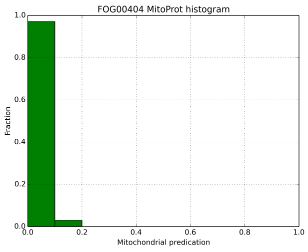

Raw data

Phobius transmembrane predictions
24 genes with posterior transmembrane prediction > 50%

FOG00405
EOG8HQC0H
ACS2
sce:ACS2

Genes: 26

Protein description
Acetyl-coA synthetase isoform expressed with fermentable carbon sources

Parent
paralog:FOG00404

SGD Description
Acetyl-coA synthetase isoform; along with Acs1p, acetyl-coA synthetase isoform is the nuclear source of acetyl-coA for histone acetylation; mutants affect global transcription; required for growth on glucose; expressed under anaerobic conditions

References

Frenkel EP, *et al*. (1977 Jan 25). Purification and properties of acetyl coenzyme A synthetase from bakers' yeast.

Midelfort CF, *et al*. (1978 Oct 25). The stereochemical course of acetate activation by yeast acetyl-CoA synthetase.

De Virgilio C, *et al*. (1992 Dec). Cloning and disruption of a gene required for growth on acetate but not on ethanol: the acetyl-coenzyme A synthetase gene of Saccharomyces cerevisiae.

Van den Berg MA, *et al*. (1995 Aug 1). ACS2, a Saccharomyces cerevisiae gene encoding acetyl-coenzyme A synthetase, essential for growth on glucose.

Kratzer S, *et al*. (1995 Aug 8). Carbon source-dependent regulation of the acetyl-coenzyme A synthetase-encoding gene ACS1 from Saccharomyces cerevisiae.

van den Berg MA, *et al*. (1996 Nov 15). The two acetyl-coenzyme A synthetases of Saccharomyces cerevisiae differ with respect to kinetic properties and transcriptional regulation.

de Jong-Gubbels P, *et al*. (1997 Aug 1). The Saccharomyces cerevisiae acetyl-coenzyme A synthetase encoded by the ACS1 gene, but not the ACS2-encoded enzyme, is subject to glucose catabolite inactivation.

[Lodi T, *et al*. (2001 Sep)](). Three target genes for the transcriptional activator Cat8p of Kluyveromyces lactis: acetyl coenzyme A synthetase genes KlACS1 and KlACS2 and lactate permease gene KlJEN1.

Peng J, *et al*. (2003 Aug). A proteomics approach to understanding protein ubiquitination.

Zeeman AM, *et al*. (2003 Jan 15). The acetyl co-enzyme A synthetase genes of Kluyveromyces lactis.

Takahashi H, *et al*. (2006 Jul 21). Nucleocytosolic acetyl-coenzyme a synthetase is required for histone acetylation and global transcription.

Mitochondrial localization predications

Predotar

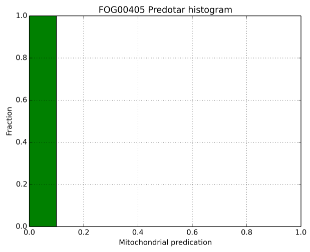

TargetP

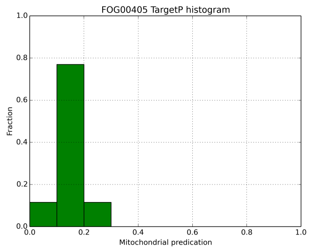

MitoProt

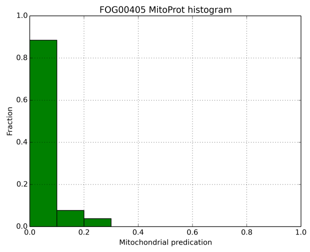

Raw data

Phobius transmembrane predictions

16 genes with posterior transmembrane prediction > 50%

FOG00406
EOG8HQC0H
ACS3
sce:absent

Genes: 3

Protein description
Uncharacterized acetyl-coA synthetase paralog

Parent
paralog:FOG00404

AspGD Description
Putative acetyl-CoA synthase

References

Zhang YQ, *et al*. (2004 Oct). Connection of propionyl-CoA metabolism to polyketide biosynthesis in Aspergillus nidulans.

Salazar M, *et al*. (2009 Dec). Uncovering transcriptional regulation of glycerol metabolism in Aspergilli through genome-wide gene expression data analysis.

Flipphi M, *et al*. (2009 Mar). Biodiversity and evolution of primary carbon metabolism in Aspergillus nidulans and other Aspergillus spp.

Lee MK, *et al*. (2014 May). NsdD is a key repressor of asexual development in Aspergillus nidulans.

Mitochondrial localization predications

[Predotar](#) [TargetP](#) [MitoProt](#)

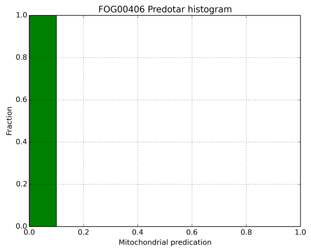

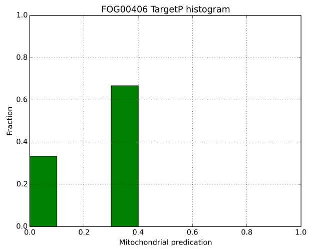

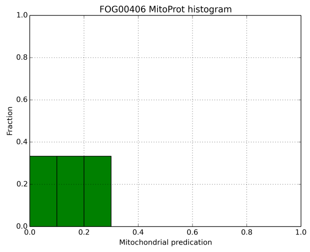

[Raw data](#)

[Phobius transmembrane predictions](#)

3 genes with posterior transmembrane prediction > 50%

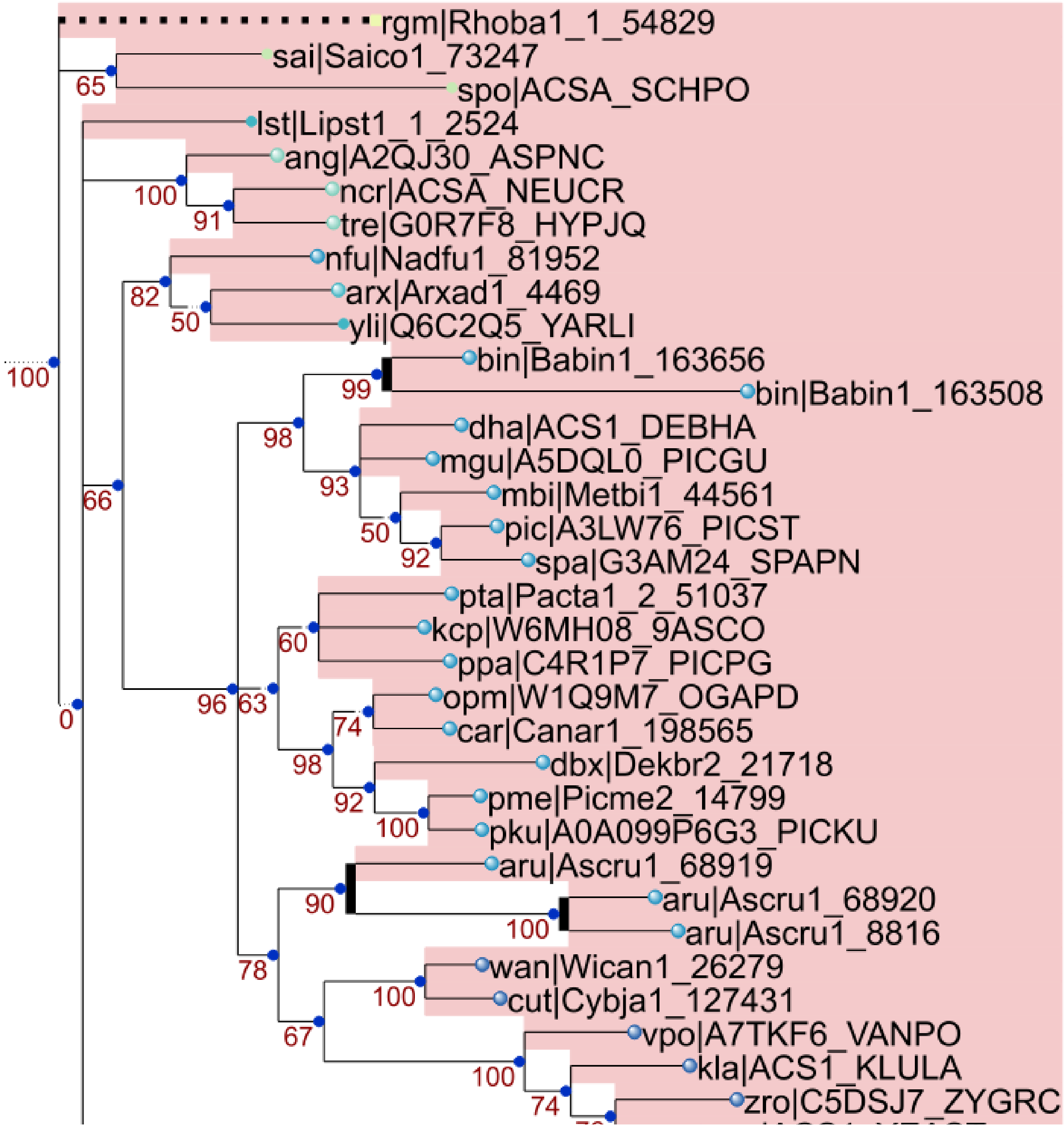

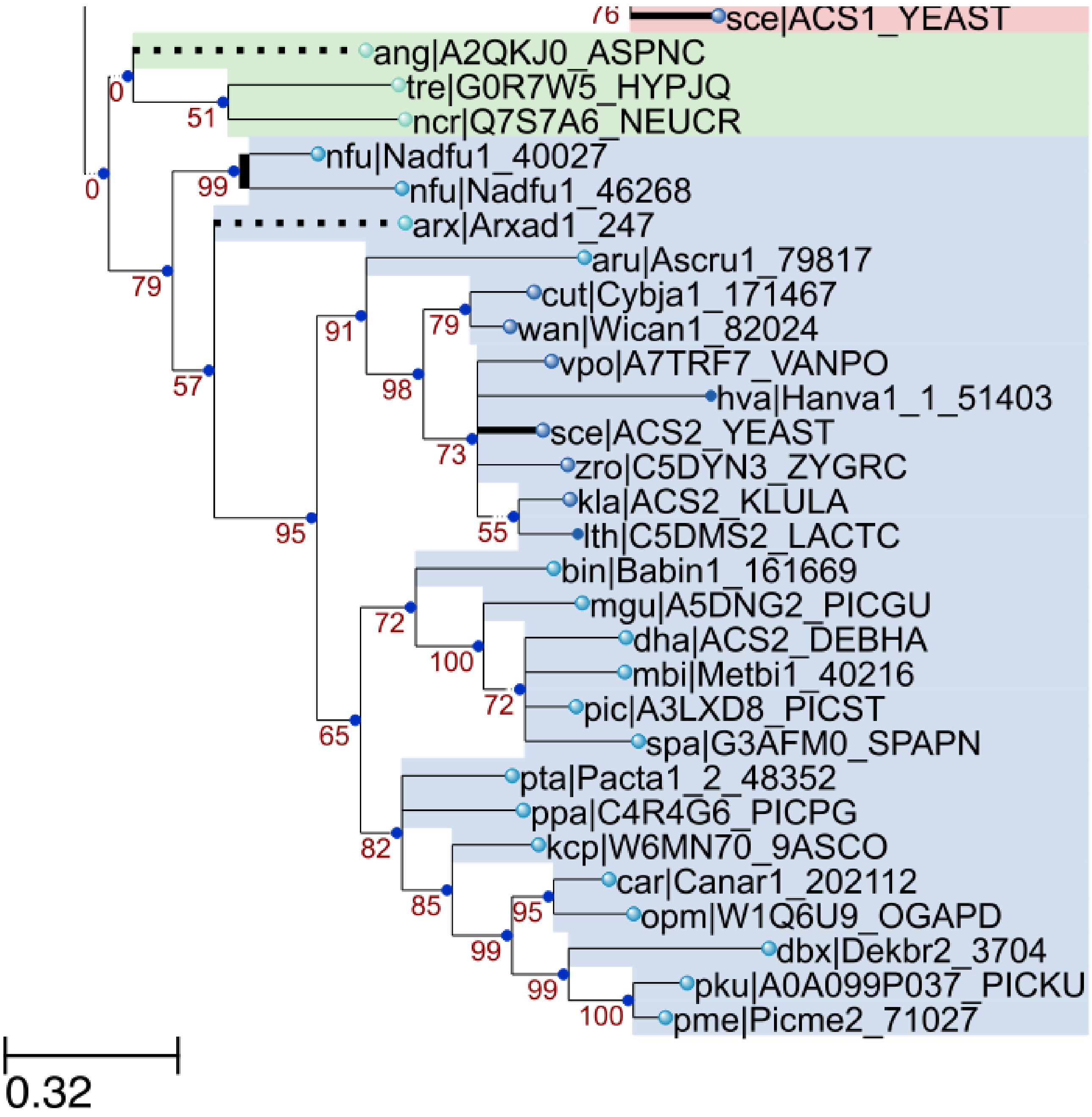

76
sce|ACS1_YEAST
ang|A2QKJ0_ASPNC
tre|G0R7W5_HYPJQ
ncr|Q7S7A6_NEUCR
nfu|Nadfu1_40027
nfu|Nadfu1_46268
arx|Arxad1_247
aru|Ascru1_79817
cut|Cybja1_171467
wan|Wican1_82024
vpo|A7TRF7_VANPO
hva|Hanva1_1_51403
sce|ACS2_YEAST
zro|C5DYN3_ZYGRC
kla|ACS2_KLULA
lth|C5DMS2_LACTC
bin|Babin1_161669
mgu|A5DNG2_PICGU
dha|ACS2_DEBHA
mbi|Metbi1_40216
pic|A3LXD8_PICST
spa|G3AFM0_SPAPN
pta|Pacta1_2_48352
ppa|C4R4G6_PICPG
kcp|W6MN70_9ASCO
car|Canar1_202112
opm|W1Q6U9_OGAPD
dbx|Dekbr2_3704
pku|A0A099P037_PICKU
pme|Picme2_71027
0
51
0
99
79
57
91
79
98
73
55
95
72
100
72
65
82
85
95
99
99
100
0.32

Analyzing Yeasts by Reconstructing Ancestry of Homologs

Home

HOG00398 FMC1 family
HOG00399
HOG00403 SUN family

FASTA
MAFFT sequence alignment
Phyml trees
Gblocks
Phobius predictions

FOG00837
EOG8P8D18
NDI0
sce:absent

Genes: 6

Protein description
Internal NADH dehydrogenase, orthologous to NDH2 in E. coli

Features
NADH

AspGD Description
Ortholog(s) have NADPH dehydrogenase activity, role in spore germination and mitochondrial inner membrane localization

References

Dinamarco TM, *et al*. (2010 Dec). The roles played by Aspergillus nidulans apoptosis-inducing factor (AIF)-like mitochondrial oxidoreductase (AifA) and NADH-ubiquinone oxidoreductases (NdeA-B and NdiA) in farnesol resistance.

Dinamarco TM, *et al*. (2011 Oct). Farnesol-induced cell death in the filamentous fungus Aspergillus nidulans.

Mitochondrial localization predications

Predotar

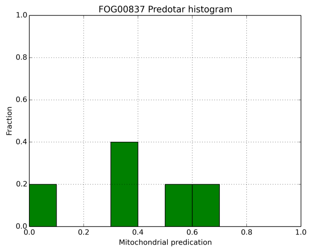

TargetP

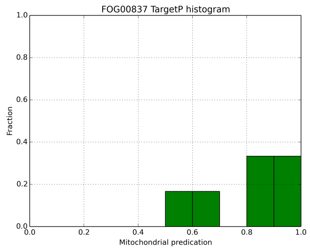

MitoProt

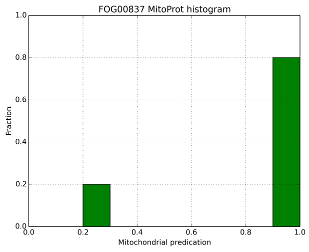

Raw data

Mitochondrial localization predications

[Phobius transmembrane predictions]

2 genes with posterior transmembrane prediction > 50%

FOG00838
EOG8P8D18
NDE0
sce:absent

Genes: 22

Protein description
External NADH and NADPH dehydrogenase

Features
NADH;NADPH

Parent
paralog:FOG00837

AspGD Description
NADH-ubiquinone oxidoreductase; respiratory-chain NADH dehydrogenase

Mitochondrial localization predications

Predotar | TargetP | MitoProt

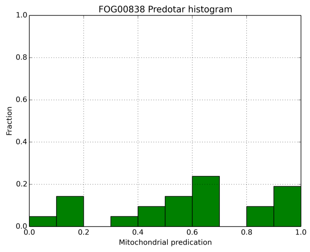

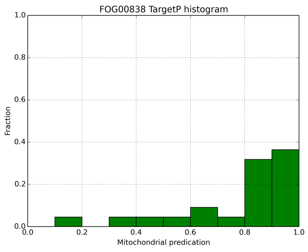

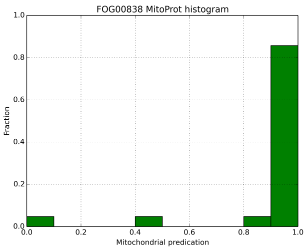

Raw data

Phobius transmembrane predictions

21 genes with posterior transmembrane prediction > 50%

FOG00839
EOG8P8D18
NDE1
sce:absent

Genes: 33

Protein description
External NADH and NADPH dehydrogenase, spo ortholog may have translate sites than enable internal NADH dehydrogenase

Features
NADH;NADPH

Parent
paralog:FOG00838

PomBase Description

mitochondrial NADH dehydrogenase (ubiquinone) Nde2 (predicted)

AspGD Description
NADH-ubiquinone oxidoreductase; respiratory-chain NADH dehydrogenase

References

Kerscher SJ, *et al.* (1999 Jul). A single external enzyme confers alternative NADH:ubiquinone oxidoreductase activity in Yarrowia lipolytica.

Todd BL, *et al.* (2006 Apr). Sterol regulatory element binding protein is a principal regulator of anaerobic gene expression in fission yeast.

Harris SD, *et al.* (2009 Mar). Morphology and development in Aspergillus nidulans: a complex puzzle.

Dinamarco TM, *et al.* (2010 Dec). The roles played by Aspergillus nidulans apoptosis-inducing factor (AIF)-like mitochondrial oxidoreductase (AifA) and NADH-ubiquinone oxidoreductases (NdeA-B and NdiA) in farnesol resistance.

Arita Y, *et al.* (2011 May). Microarray-based target identification using drug hypersensitive fission yeast expressing ORFeome.

Dinamarco TM, *et al.* (2011 Oct). Farnesol-induced cell death in the filamentous fungus Aspergillus nidulans.

Lando D, *et al.* (2012). The S. pombe histone H2A dioxygenase Ofd2 regulates gene expression during hypoxia.

Carpy A, *et al.* (2014 Aug). Absolute proteome and phosphoproteome dynamics during the cell cycle of Schizosaccharomyces pombe (Fission Yeast).

Guo L, *et al.* (2016 Oct 13). Global Fitness Profiling Identifies Arsenic and Cadmium Tolerance Mechanisms in Fission Yeast.

Dudin O, *et al.* (2017 Apr). A systematic screen for morphological abnormalities during fission yeast sexual reproduction identifies a mechanism of actin aster formation for cell fusion.

Lee J, *et al.* (2017 Feb 20). Chromatin remodeller Fun30^{Fft3} induces nucleosome disassembly to facilitate RNA polymerase II elongation.

Mitochondrial localization predications

Predotar | TargetP | MitoProt

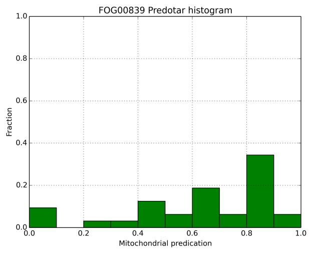

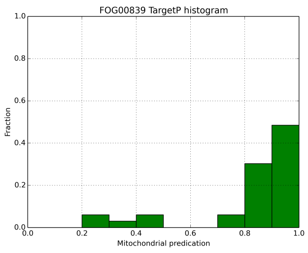

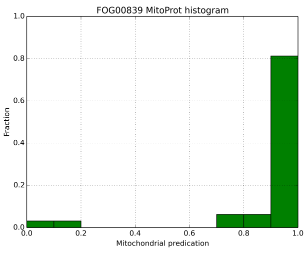

Raw data

Phobius transmembrane predictions
31 genes with posterior transmembrane prediction > 50%

FOG00840
EOG8P8D18
NDE1.2
sce:absent

Genes: 1

Protein description
External NADH and NADPH dehydrogenase, spo ortholog may have translate sites than enable internal NADH dehydrogenase

Parent
paralog:FOG00839

PomBase Description
mitochondrial NADH dehydrogenase (ubiquinone) Nde1 (predicted)

Mitochondrial localization predications

Predotar | TargetP | MitoProt

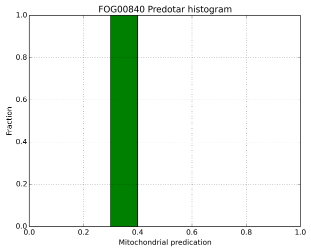

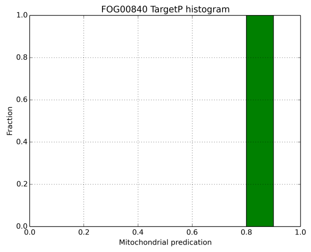

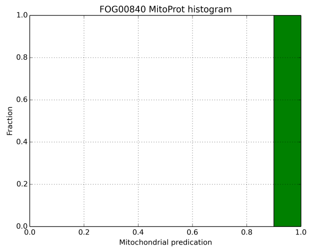

Raw data

Phobius transmembrane predictions
1 genes with posterior transmembrane prediction > 50%

FOG00841
EOG8P8D18
NDE1
sce:NDE1

Genes: 2

Protein description
External NADH dehydrogenase, loss of NADPH activity

Parent
ortholog:FOG00839

SGD Description
Mitochondrial external NADH dehydrogenase; type II NAD(P)H:quinone oxidoreductase that catalyzes the oxidation of cytosolic NADH; Nde1p and Nde2p provide cytosolic NADH to the mitochondrial respiratory chain; NDE1 has a paralog, NDE2, that arose from the whole genome duplication

References

Small WC, *et al*. (1998 Aug). Identification of a cytosolically directed NADH dehydrogenase in mitochondria of Saccharomyces cerevisiae.

Luttik MA, *et al*. (1998 Sep 18). The Saccharomyces cerevisiae NDE1 and NDE2 genes

encode separate mitochondrial NADH dehydrogenases catalyzing the oxidation of cytosolic NADH.

Kerscher SJ, *et al*. (2000 Aug 15). Diversity and origin of alternative NADH:ubiquinone oxidoreductases.

Overkamp KM, *et al*. (2000 May). In vivo analysis of the mechanisms for oxidation of cytosolic NADH by Saccharomyces cerevisiae mitochondria.

Joseph-Horne T, *et al*. (2001 Apr 2). Fungal respiration: a fusion of standard and alternative components.

Grandier-Vazeille X, *et al*. (2001 Aug 21). Yeast mitochondrial dehydrogenases are associated in a supramolecular complex.

Davidson JF, *et al*. (2001 Dec). Mitochondrial respiratory electron carriers are involved in oxidative stress during heat stress in Saccharomyces cerevisiae.

Bakker BM, *et al*. (2001 Jan). Stoichiometry and compartmentation of NADH metabolism in Saccharomyces cerevisiae.

Påhlman IL, *et al*. (2002 Aug 2). Kinetic regulation of the mitochondrial glycerol-3-phosphate dehydrogenase by the external NADH dehydrogenase in Saccharomyces cerevisiae.

Sickmann A, *et al*. (2003 Nov 11). The proteome of Saccharomyces cerevisiae mitochondria.

Mitochondrial localization predications

Predotar | TargetP | MitoProt

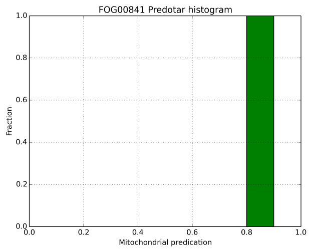

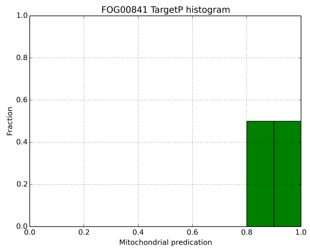

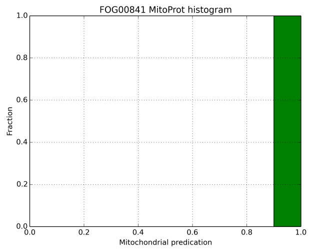

Raw data

Phobius transmembrane predictions
2 genes with posterior transmembrane prediction > 50%

FOG00842

NDI2
sce:absent

Genes: 1

Features
NADH

Parent
paralog:FOG00839

Mitochondrial localization predications

Predotar | TargetP | MitoProt

Mitochondrial localization predications

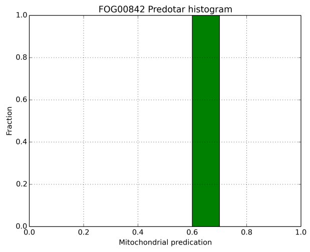

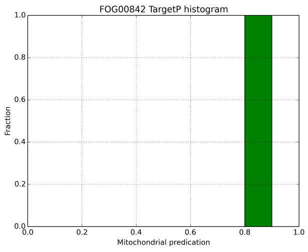

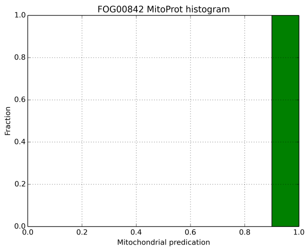

Raw data

Phobius transmembrane predictions
0 genes with posterior transmembrane prediction > 50%

FOG00843

NDE1.3
sce:absent

Genes: 2

Protein description
External NADH and NADPH dehydrogenase

Parent
paralog:FOG00841

Mitochondrial localization predications

Predotar TargetP MitoProt

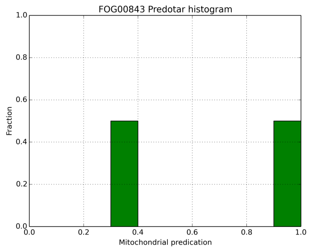

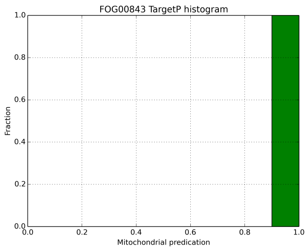

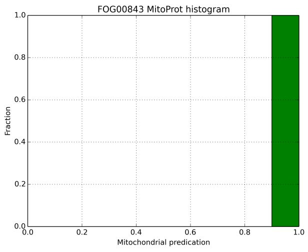

Raw data

Phobius transmembrane predictions
2 genes with posterior transmembrane prediction > 50%

FOG00844

NDE1.4
sce:absent

Genes: 1

Protein description
External NADH and NADPH dehydrogenase

Parent
paralog:FOG00843

Mitochondrial localization predications

Predotar TargetP MitoProt

Mitochondrial localization predications

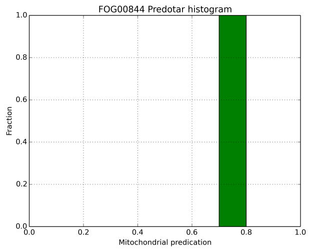

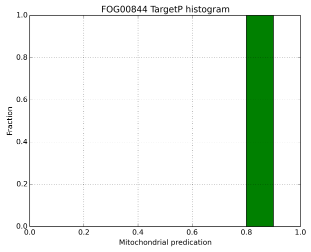

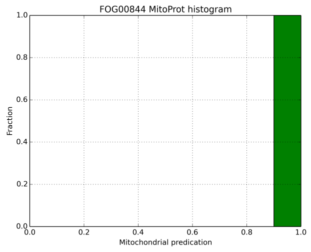

Raw data

Phobius transmembrane predictions
1 genes with posterior transmembrane prediction > 50%

FOG00845
EOG8P8D18
NDE2
sce:NDE2

Genes: 1

Protein description
External NADH dehydrogenase

Features
NADH

Parent
ohnolog:FOG00841

SGD Description
Mitochondrial external NADH dehydrogenase; catalyzes the oxidation of cytosolic NADH; Nde1p and Nde2p are involved in providing the cytosolic NADH to the mitochondrial respiratory chain; NDE2 has a paralog, NDE1, that arose from the whole genome duplication

References

Zhu QS, *et al*. (1988 Jan 5). Direct interaction between yeast NADH-ubiquinone oxidoreductase, succinate-ubiquinone oxidoreductase, and ubiquinol-cytochrome c oxidoreductase in the reduction of exogenous quinones.

Shankar CS, *et al*. (1996 Sep). Mitochondrial NADH dehydrogenase activity and ability to tolerate acetaldehyde determine faster ethanol production in Saccharomyces cerevisiae.

Luttik MA, *et al*. (1998 Sep 18). The Saccharomyces cerevisiae NDE1 and NDE2 genes encode separate mitochondrial NADH dehydrogenases catalyzing the oxidation of cytosolic NADH.

Kerscher SJ, *et al*. (2000 Aug 15). Diversity and origin of alternative NADH:ubiquinone oxidoreductases.

Overkamp KM, *et al*. (2000 May). In vivo analysis of the mechanisms for oxidation of cytosolic NADH by Saccharomyces cerevisiae mitochondria.

Grandier-Vazeille X, *et al*. (2001 Aug 21). Yeast mitochondrial dehydrogenases are associated in a supramolecular complex.

Davidson JF, *et al*. (2001 Dec). Mitochondrial respiratory electron carriers are involved in oxidative stress during heat stress in Saccharomyces cerevisiae.

Bakker BM, *et al*. (2001 Jan). Stoichiometry and compartmentation of NADH metabolism in Saccharomyces cerevisiae.

Påhlman IL, *et al*. (2002 Aug 2). Kinetic regulation of the mitochondrial glycerol-3-phosphate dehydrogenase by the external NADH dehydrogenase in Saccharomyces cerevisiae.

Sickmann A, *et al*. (2003 Nov 11). The proteome of Saccharomyces cerevisiae mitochondria.

Mitochondrial localization predications

Predotar

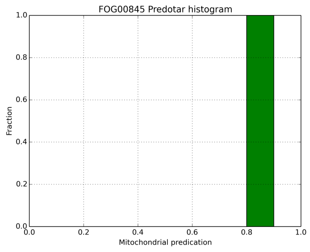

TargetP

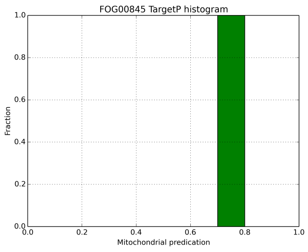

MitoProt

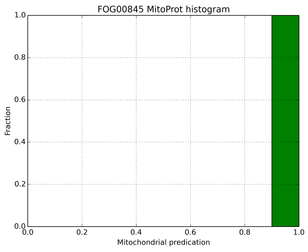

Raw data

Phobius transmembrane predictions
1 genes with posterior transmembrane prediction > 50%

FOG00846
EOG8P8D18
NDI1
sce:NDI1

Genes: 9

Protein description
Internal NADH dehydrogenase

Features
NADH

Parent
paralog:FOG00839

SGD Description
NADH:ubiquinone oxidoreductase; transfers electrons from NADH to ubiquinone in the respiratory chain but does not pump protons, in contrast to the higher eukaryotic multisubunit respiratory complex I; phosphorylated; involved in Mn and H2O2 induced apoptosis; upon apoptotic stress, Ndip is activated in the mitochondria by N-terminal cleavage, and the truncated protein translocates to the cytoplasm to induce apoptosis; homolog of human AMID

References

De Vries S, *et al*. (1992 Feb 1). Primary structure and import pathway of the rotenone-insensitive NADH-ubiquinone oxidoreductase of mitochondria from Saccharomyces cerevisiae.

Small WC, *et al.* (1998 Aug). Identification of a cytosolically directed NADH dehydrogenase in mitochondria of Saccharomyces cerevisiae.

Luttik MA, *et al.* (1998 Sep 18). The Saccharomyces cerevisiae NDE1 and NDE2 genes encode separate mitochondrial NADH dehydrogenases catalyzing the oxidation of cytosolic NADH.

Grandier-Vazeille X, *et al.* (2001 Aug 21). Yeast mitochondrial dehydrogenases are associated in a supramolecular complex.

Reinders J, *et al.* (2007 Nov). Profiling phosphoproteins of yeast mitochondria reveals a role of phosphorylation in assembly of the ATP synthase.

Renvoisé M, *et al.* (2014 Jun 25). Quantitative variations of the mitochondrial proteome and phosphoproteome during fermentative and respiratory growth in Saccharomyces cerevisiae.

Mitochondrial localization predications

Predotar | TargetP | MitoProt

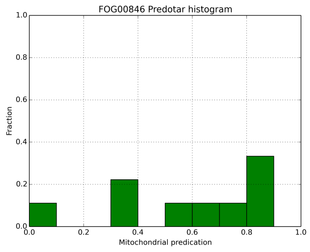

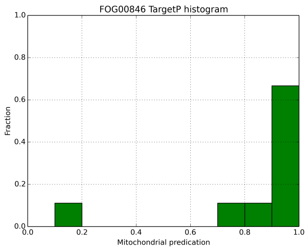

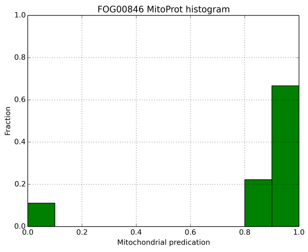

Raw data

Phobius transmembrane predictions

9 genes with posterior transmembrane prediction > 50%

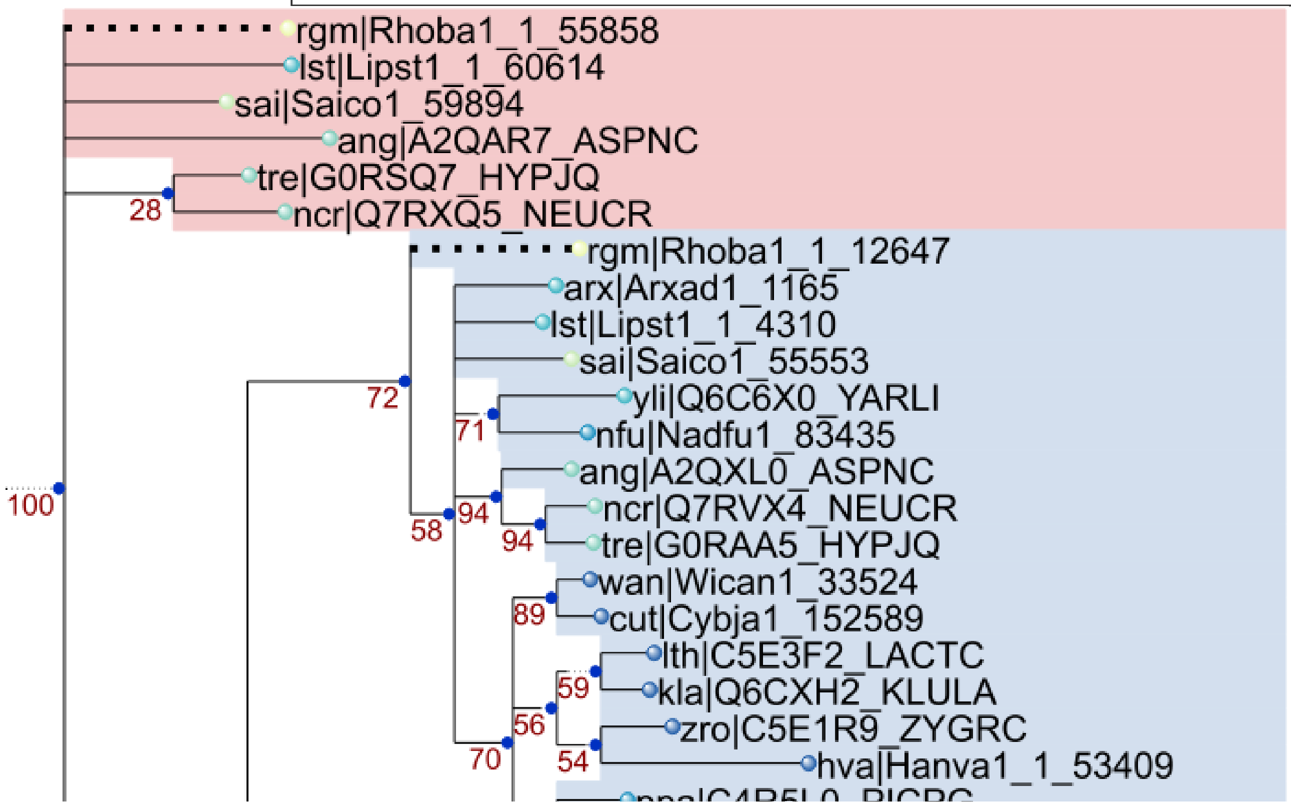

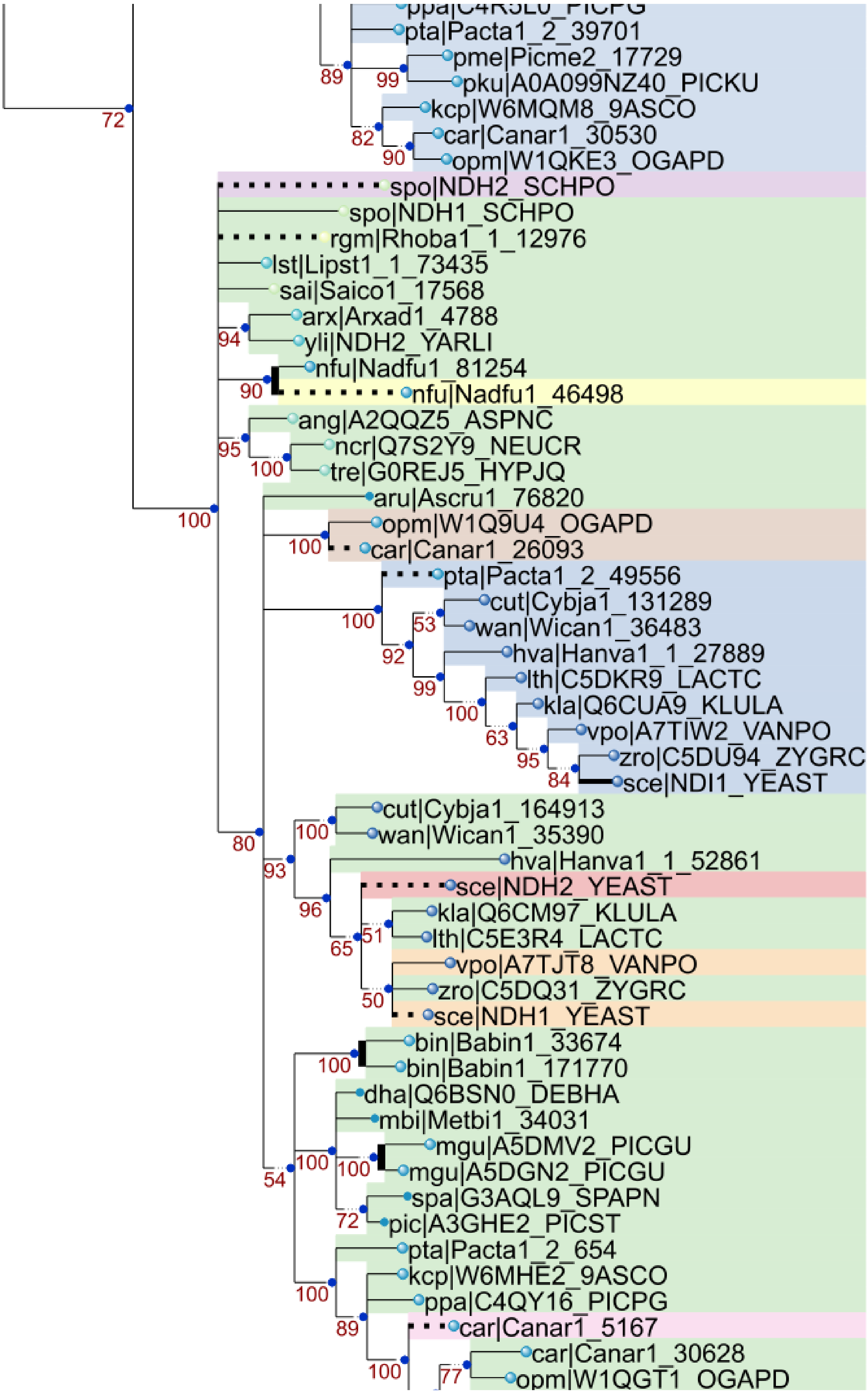
pta|Pacta1_2_39701
pme|Picme2_17729
pku|A0A099NZ40_PICKU
kcp|W6MQM8_9ASCO
car|Canar1_30530
opm|W1QKE3_OGAPD
spo|NDH2_SCHPO
spo|NDH1_SCHPO
rgm|Rhoba1_1_12976
lst|Lipst1_1_73435
sai|Saico1_17568
arx|Arxad1_4788
yli|NDH2_YARLI
nfu|Nadfu1_81254
nfu|Nadfu1_46498
ang|A2QQZ5_ASPNC
ncr|Q7S2Y9_NEUCR
tre|G0REJ5_HYPJQ
aru|Ascru1_76820
opm|W1Q9U4_OGAPD
car|Canar1_26093
pta|Pacta1_2_49556
cut|Cybja1_131289
wan|Wican1_36483
hva|Hanva1_1_27889
lth|C5DKR9_LACTC
kla|Q6CUA9_KLULA
vpo|A7TIW2_VANPO
zro|C5DU94_ZYGRC
sce|NDI1_YEAST
cut|Cybja1_164913
wan|Wican1_35390
hva|Hanva1_1_52861
sce|NDH2_YEAST
kla|Q6CM97_KLULA
lth|C5E3R4_LACTC
vpo|A7TJT8_VANPO
zro|C5DQ31_ZYGRC
sce|NDH1_YEAST
bin|Babin1_33674
bin|Babin1_171770
dha|Q6BSN0_DEBHA
mbi|Metbi1_34031
mgu|A5DMV2_PICGU
mgu|A5DGN2_PICGU
spa|G3AQL9_SPAPN
pic|A3GHE2_PICST
pta|Pacta1_2_654
kcp|W6MHE2_9ASCO
ppa|C4QY16_PICPG
car|Canar1_5167
car|Canar1_30628
opm|W1QGT1_OGAPD
72
89
99
82
90
94
90
95
100
100
100
100
53
92
99
100
63
95
84
80
100
93
96
65
51
50
100
54
100
100
72
100
89
100
77

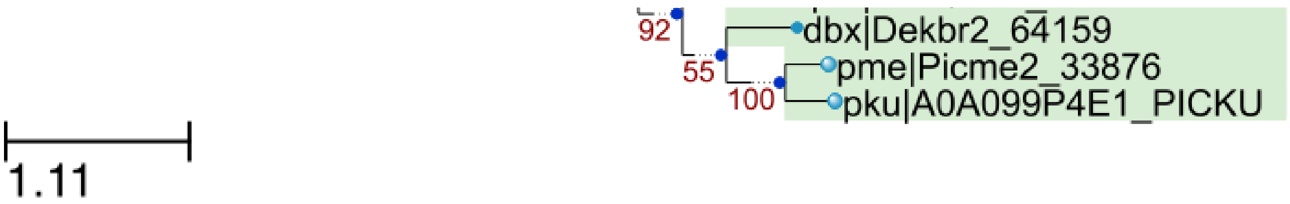
92
55
100
dbx|Dekbr2_64159
pme|Picme2_33876
pku|A0A099P4E1_PICKU
1.11

References

Patrícia Carneiro, Margarida Duarte, and Arnaldo Videira. The external alternative NAD(P)H dehydrogenase NDE3 is localized both in the mitochondria and in the cytoplasm of *Neurospora crassa*. *Journal of Molecular Biology*, 368(4):1114–1121, 2007.
