# Supplementary figures and images for: AYbRAH: a curated ortholog database for yeasts and fungi spanning 600 million years of evolution
Source: Database (Oxford). 2019 Mar 20;2019:baz022. doi: 10.1093/database/baz022 (PMC6425859; doi:10.1093/database/baz022)

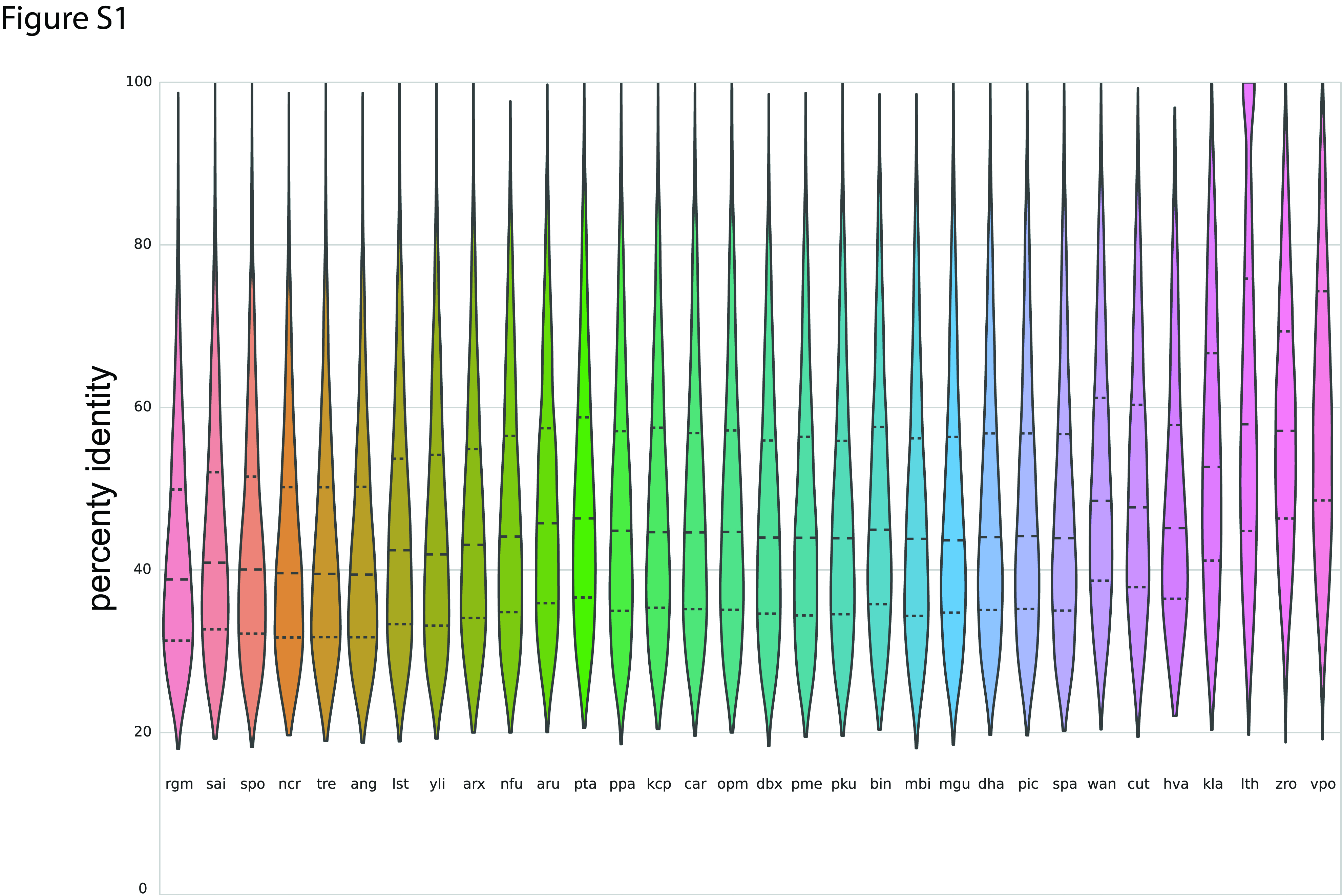

Supplement: supp_fig1_oid_by_pid_baz022 [file supp_fig1_oid_by_pid_baz022.jpeg]

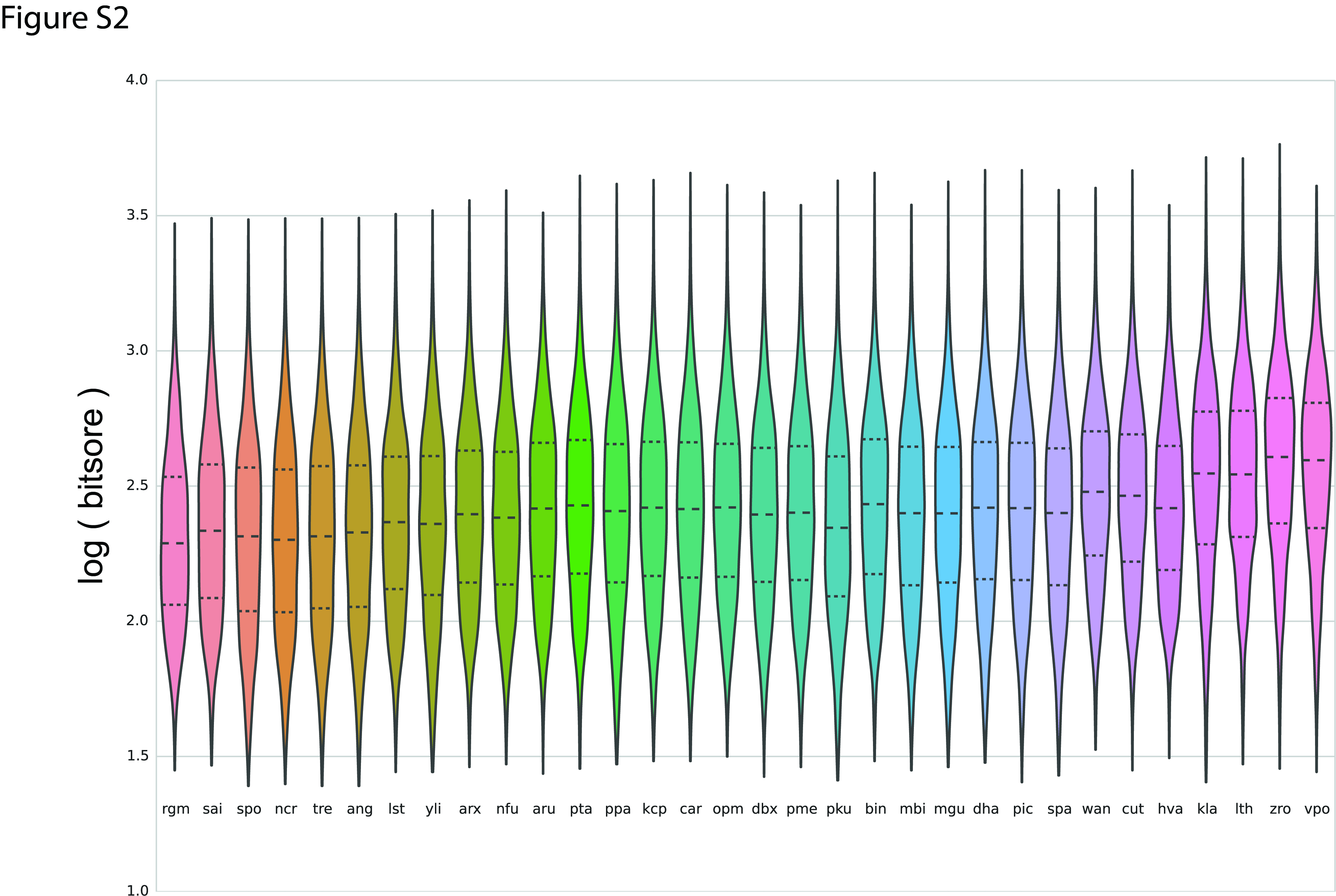

Supplement: supp_fig2_oid_score_log10_baz022 [file supp_fig2_oid_score_log10_baz022.jpeg]

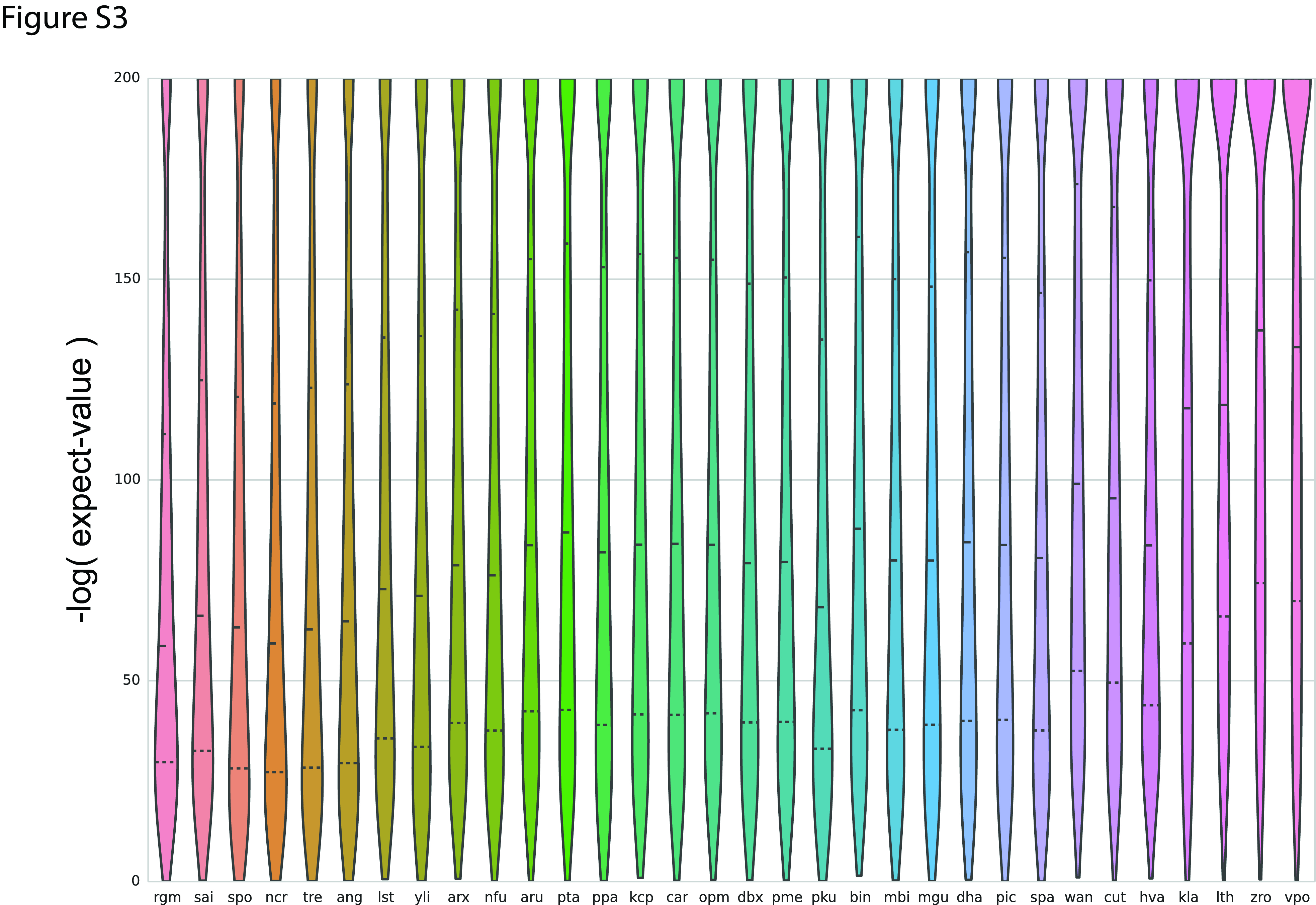

Supplement: supp_fig3_oid_evalue_neglog10_baz022 [file supp_fig3_oid_evalue_neglog10_baz022.jpeg]
